# Human Ccr4 and Caf1 Deadenylases Regulate Proliferation and Tumorigenicity of Human Gastric Cancer Cells via Modulating Cell Cycle Progression

**DOI:** 10.3390/cancers13040834

**Published:** 2021-02-17

**Authors:** Xiao-Hui Song, Xiao-Yan Liao, Xu-Ying Zheng, Jia-Qian Liu, Zhe-Wei Zhang, Li-Na Zhang, Yong-Bin Yan

**Affiliations:** 1 State Key Laboratory of Membrane Biology, School of Life Sciences, Tsinghua University, Beijing 100084, China; songxh2018@mail.tsinghua.edu.cn (X.-H.S.); liaoxy16@mails.tsinghua.edu.cn (X.-Y.L.); zhengxy20@mails.tsinghua.edu.cn (X.-Y.Z.); liujq19@mails.tsinghua.edu.cn (J.-Q.L.); zzw19@mails.tsinghua.edu.cn (Z.-W.Z.); 2 College of Life Science and Chemistry, Faculty of Environment and Life, Beijing University of Technology, Beijing 100124, China

**Keywords:** Caf1, Ccr4, Ccr4-Not complex, cell cycle progression, deadenylase, mRNA decay, p21, processing body, stomach adenocarcinoma

## Abstract

**Simple Summary:**

Cancer cells generally reprogram their gene expression profiles to satisfy continuous growth, proliferation, and metastasis. Most eukaryotic mRNAs are degraded in a deadenylation-dependent pathway, in which deadenylases are the key enzymes. We found that human Ccr4 (hCcr4a/b) and Caf1 (hCaf1a/b), the dominant cytosolic deadenylases, were dysregulated in several types of cancers including stomach adenocarcinoma. Stably knocking down hCaf1a/b or hCcr4a/b blocks cell cycle progression by enhancing the levels of cell cycle inhibitors and by inhibiting the formation of processing bodies, which are cytosolic foci involved in mRNA metabolism. More importantly, depletion of hCaf1a/b or hCcr4a/b dramatically inhibits cell proliferation and tumorigenicity. Our results suggest that perturbating global RNA metabolism may provide a potential novel strategy for cancer treatment.

**Abstract:**

Cancer cells generally have reprogrammed gene expression profiles to meet the requirements of survival, continuous division, and metastasis. An interesting question is whether the cancer cells will be affected by interfering their global RNA metabolism. In this research, we found that human Ccr4a/b (hCcr4a/b) and Caf1a/b (hCaf1a/b) deadenylases, the catalytic components of the Ccr4-Not complex, were dysregulated in several types of cancers including stomach adenocarcinoma. The impacts of the four deadenylases on cancer cell growth were studied by the establishment of four stable MKN28 cell lines with the knockdown of hCcr4a/b or hCaf1a/b or transient knockdown in several cell lines. Depletion of hCcr4a/b or hCaf1a/b significantly inhibited cell proliferation and tumorigenicity. Mechanistic studies indicated that the cells were arrested at the G2/M phase by knocking down hCaf1a, while arrested at the G0/G1 phase by depleting hCaf1b or hCcr4a/b. The four enzymes did not affect the levels of CDKs and cyclins but modulated the levels of CDK–cyclin inhibitors. We identified that hCcr4a/b, but not hCaf1a/b, targeted the *p21* mRNA in the MKN28 cells. Furthermore, depletion of any one of the four deadenylases dramatically impaired processing-body formation in the MKN28 and HEK-293T cells. Our results highlight that perturbating global RNA metabolism may severely affect cancer cell proliferation, which provides a potential novel strategy for cancer treatment.

## 1. Introduction

The eight hallmarks of cancer comprise limitless replication, avoidance of cell death, sustaining proliferation, insensitivity to growth suppressors, inducing angiogenesis, activation of invasion and metastasis, evasion of immune destruction, and metabolic reprogramming [1,2]. These hallmarks are acquired from the intrinsic instability of a cancer cell genome and the reprogrammed gene expression profile to satisfy cancer cell proliferation and survival. In normal cells, cell cycle progression is orchestrated by cyclin-dependent kinases (CDKs) and various CDK regulators including cyclins respond to both extracellular and intracellular signals [3,4]. To support the sustaining proliferation, cancer cells need to constantly turn on or amplify the factors promoting cell cycle progression, whereas they turn off or downregulate the cell cycle inhibitors and cell death inducers [4]. In addition to acting as the driving force of cell cycle progression, CDKs and their regulators have recently been found to play crucial roles in other cellular processes such as transcriptional regulation, as well as metabolic and signaling pathways [5,6,7,8,9], implying that the hallmarks of cancer result from a coordinated action of diverse factors leading to alterations in the gene expression profile.

Gene expression can be regulated by multiple levels, including transcription, post-transcriptional modifications, and translation. The number of translationally active transcripts is positively regulated by transcription and negatively regulated by mRNA decay. In eukaryotes, most mRNAs are degraded in a deadenylation-dependent manner. Deadenylation is achieved by deadenylases, which are a group of 3′ to 5′ exoribonucleases with a high substrate preference of poly(A) [10,11]. Among them, deadenylases in the PAN2–PAN3 and Ccr4–Not complexes are considered to be responsible for global deadenylation, and thereafter the decay of most mRNAs in the cytosol [12,13], while poly(A)-specific ribonucleases (PARN) have been proposed to contribute to the selective degradation of highly regulated mRNAs and the biogenesis of a number of small non-coding RNAs [10,14]. In addition to the basic functions in mRNA decay, deadenylases have been found to participate in diverse cellular events including proliferation, cell death, DNA-damage response, and genome stability of the cells [14,15,16], which are also important to carcinogenesis. Actually, dysregulation of deadenylases in the Ccr4–Not complex or PARN has been observed in several types of cancers [17,18,19,20,21,22,23].

Theoretically, there are two possible means for deadenylases to modulate cell proliferation as follows: degradation of transcripts encoding proliferation-promoting proteins via its deadenylation activity or accumulation of transcripts encoding proliferation-inhibiting proteins via the inhibition of its activity by antiproliferation proteins. For the first one, PARN has been identified to trigger the decay of transcripts encoding tumor suppressors including p53 [24], p21 [17], GADD45α [25], nucleophosmin [26], MDM2 [27], c-fos, TNFα [28], c-myc [29], and the biogenesis of miRNAs targeting the *p53* mRNA [30,31]. Components of the Ccr4–Not complex (NOT proteins) are involved in the cell cycle progression or cell death by modulating the decay of transcripts encoding p53 [32,33], Atg7 [15], p27 [34], insulin-like growth factor binding protein-5 (IGFBP-5) [33], receptor-interacting protein kinase 1 (RIPK1), and RIPK3 [35] in vertebrates and maternal cyclin A and B in fly oocytes [36,37]. As for the action of deadenylase regulators, the activities of PARN and the Ccr4–Not complex can be modulated by many RNA-binding proteins [10,14]. In particular, the Ccr4–Not complex modulates the antiproliferative function of the BTG/Tob family proteins [38,39,40,41,42,43].

Despite the crucial roles of deadenylases in cell proliferation and cell death, little is known about the roles of deadenylases in carcinogenesis and the possibility of deadenylases as therapy targets. In particular, knowledge regarding the Ccr4–Not complex in cell cycle progression has mainly been discovered in yeast and flies, while its function in mammalians has not been recognized until recently. There are two types of deadenylases, Ccr4 and Caf1, in the Ccr4–Not complex. Each of which has at least two highly conserved orthologues in human cells, hCcr4a/CNOT6, hCcr4b/CNOT6L, hCaf1a/CNOT7, and hCaf1b/CNOT8, which perform overlapping and distinct roles in cellular events [44,45]. Although dissimilarities in the functions of the two pairs of isoenzymes have been observed, it is unclear whether they act differentially in cancer cell proliferation. In this study, these problems are addressed by the dissimilarities in the upregulation of the four deadenylases in gastric tumors and in the proliferation of stable cell lines with the depletion of each deadenylase. The four deadenylases, i.e., hCcr4a/b and hCaf1a/b, are highly expressed in some types of tumors. The proliferation of the MKN28 cells were inhibited differentially via the dissimilar regulation mechanisms of cell cycle progression by these four enzymes. Nonetheless, depletion of any one of the four deadenylases abolished the ability of tumorigenicity of the MKN28 cells in nude mice, suggesting that the hCcr4 and hCaf1 deadenylases might be potential targets for cancer therapy.

## 2. Results

### 2.1. The Human Ccr4 (hCcr4) and Human Caf1 (hCaf1) Deadenylases Are Dysregulated in Some Types of Tumors

To explore the potential involvement of the hCcr4 and hCaf1 deadenylases in cancers, we analyzed the RNA sequencing expression data in the TCGA and GTEx projects using GEPIA, which is an interactive web server developed by the Zhang Lab [46]. As shown in Figure S1, the four deadenylases were differentially dysregulated in some types of tumors. It is interesting that *hCaf1a* and *hCcr4a* are upregulated in tumors with abnormal expression, while both upregulation and downregulation are observed for *hCaf1b* and *hCcr4b*. Downregulation of *hCaf1b* in lung squamous cell carcinoma (LUSC) and testicular gem cell tumors (TGCT) or *hCcr4b* in uterine carcinosarcoma (UCS) is not accompanied with the upregulation of any of the other three deadenylases, suggesting that dysregulation of any one of hCcr4a/b and hCaf1a/b deadenylases are not compensated by the other deadenylases. Consistently, the expression of the four deadenylases are at different levels according to the databases, in which *hCaf1a/b* are generally expressed at a higher level than *hCcr4a/b* (Figure S2A). Furthermore, the correlation analysis of the expression between each pair of the four deadenylases indicates that the *hCcr4a*/*hCcr4b* and *hCcr4a*/*hCaf1b* pair has a correlation R value of around 0.7, while the other pair exhibits poor correlation in somatic normal tissues and tumors (Figure S2B). This implies that although hCcr4a/b and hCaf1a/b are catalytic components of the Ccr4–Not complex, the four deadenylases may perform distinct functions independent of the large complex.

**Figure 1:**
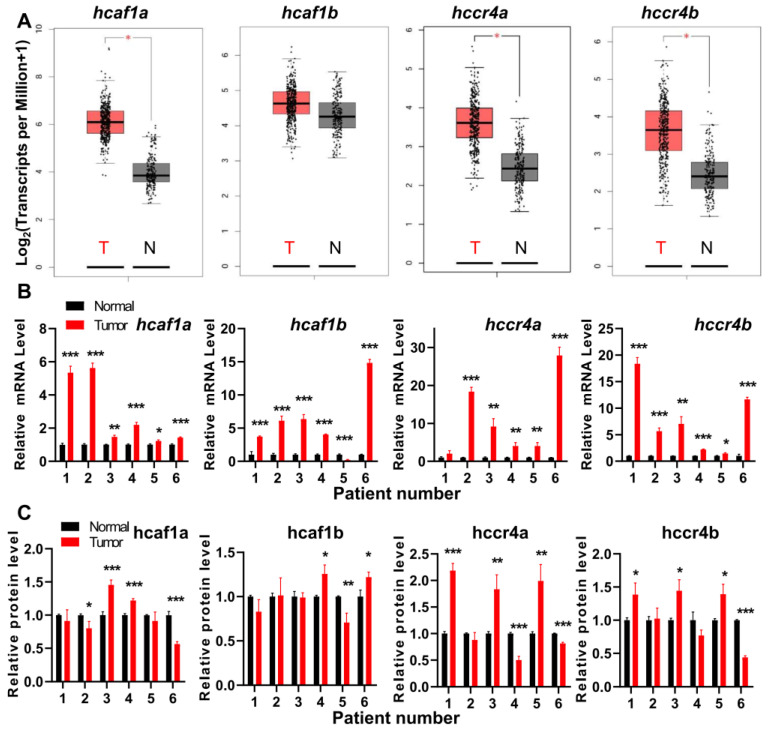
The human Ccr4 (hCcr4)a/b and human Caf1 (hCaf1)a/b deadenylases were dysregulated in gastric cancers. (**A**) Expression profiles of the four deadenylases in stomach adenocarcinoma (STAD) database analyzed by the web server GEPIA; (**B**) Changes in the mRNA levels of the four deadenylases in patient samples analyzed by qPCR using β-actin as the internal control (*n* = 3); (**C**) Changes in protein levels of the four deadenylases in patient samples analyzed by Western blot using GAPDH as the internal control (*n* = 3). All experiments were performed with three biological replicates and the data were normalized by taking the control (normal) in each replicate as 1.0. * *p* < 0.05, ** *p* < 0.01, and *** *p* < 0.001.

A close inspection of the tumors with upregulation of deadenylases indicates that all of the four deadenylases are upregulated in pancreatic adenocarcinoma (PAAD), while three, except for *hCaf1b,* are upregulated in stomach adenocarcinoma (STAD) and thymoma (THYM). The details of the dysregulation of the deadenylases in STAD database are shown in Figure 1A. Considering that the sample size of PAAD is rather small as compared with STAD, we chose gastric cancers for further verification using patient samples (Figure 1B). For all tested samples, the qPCR study indicated that the mRNA levels of most gastric tumors were upregulated for all of the four genes, although the database analysis indicated that the change of *hcaf1b* mRNA level was not significant in STAD. The differences between the in silicon and experimental analysis might be caused by the different analyzing methods and the potential influence of tumors on the paracancerous tissues. Among the four genes, the upregulation of *hCcr4a/b* was more significant with an increase up to ~20-fold. The changes in the protein levels were quite different from the mRNA levels (Figure 1C, Figures S3 and S11). Only about half of the samples had a significant increase in tumor samples, while about one-fourth was downregulated. The inconsistency of the protein levels with the mRNA levels suggested that the protein levels of the four deadenylases were modulated by multiple factors including the translation efficiency and half-life of degradation. Actually, we recently found that the inconsistency in the changes of mRNA and proteins levels of MDM2 is caused by alterations in translation efficiency modulated by PARN [27]. Unfortunately, little is known for the turnover of the Ccr4–Not complex components and further research is needed to elucidate the regulation of the Ccr4–Not complex. Nonetheless, the observations from the patient samples confirmed the results from the RNA sequencing expression database that the four deadenylases are probably dysregulated in gastric cancers.

### 2.2. Depletion of the hCcr4a/b or hCaf1a/b Deadenylases Inhibits Cell Proliferation and Tumorigenicity

**Figure 2:**
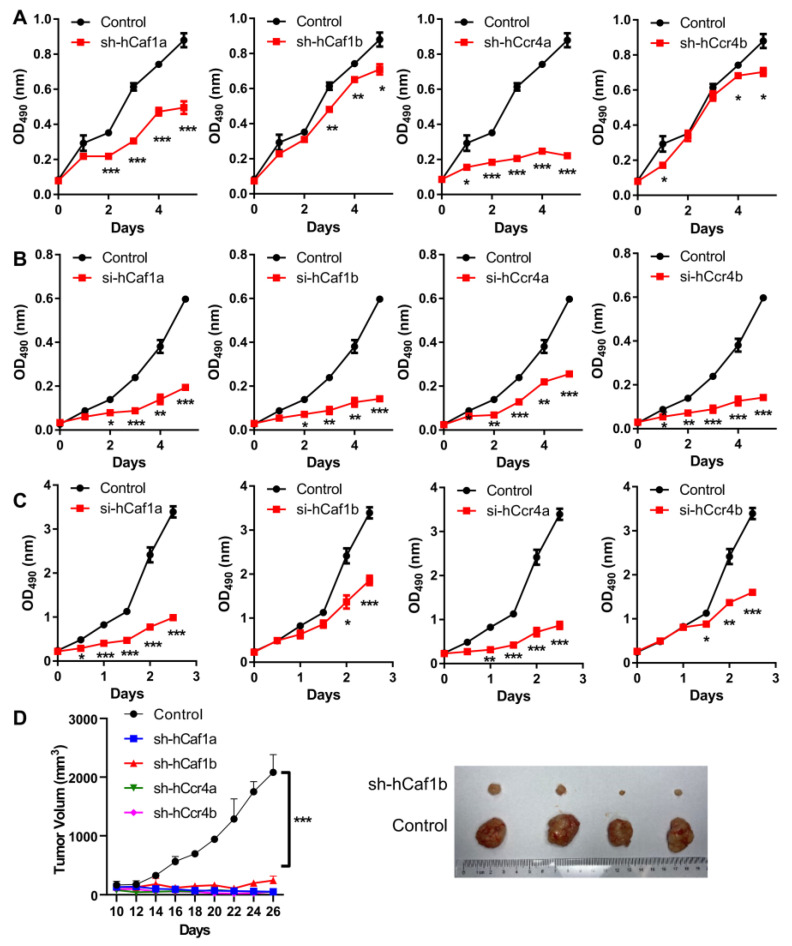
Depletion of hCcr4a/b or hCaf1a/b inhibits cell proliferation and tumorigenicity. (**A**) Growth curves of the stable knocking down MKN28 cells determined by the MTS assay (*n* = 4); (**B**) Growth curves of the AGS cells with knockdown of deadenylases by siRNA determined by the MTS assay (*n* = 3); (**C**) Growth curves of the HeLa cells with knockdown of deadenylases by siRNA determined by the MTS assay (*n* = 3); (**D**) Tumorigenicity of the MKN28 cells evaluated by the nude mice model. The left panel shows the time-course changes in tumor volume, while the right panel shows the photograph of excised xenografted tumors after inoculation for 2 months (*n* = 4). The xenografts of hCaf1a, hCcr4a, and hCcr4b did not result in tumors after inoculation for 2 months (also refer to Figure S6), and therefore only the control and sh-hCaf1b groups are presented. * *p* < 0.05, ** *p* < 0.01, and *** *p* < 0.001.

To investigate the potential functions of the four deadenylases in gastric cancer cell growth and survival, we established four stable knockdown MKN28 cell lines, with the depletion of hCcr4a/b or hCaf1a/b by shRNA (Figure S4), and the polyclones with the largest knockdown efficiencies at both mRNA and protein levels were used for further analysis (Figure S5). It is worth noting that the establishment of stable knockdown AGS cell lines were unsuccessful, which could have been caused by knockdown significantly reducing AGS cell viability. The obtained stable MKN28 cells also exhibited reduced cell viability and grew much slower than the control cells, implying that the depletion of any one of the four deadenylases might severely affect gastric cancer cell growth or death. Thus, the proliferation of the cells was studied, and the results indicated that depletion of any deadenylase inhibited MKN28 cell proliferation to dissimilar extents (Figure 2A). Among them, the effect of hCcr4a depletion was the most severe, while hCaf1b and hCcr4b depletion only produced a mild decrease after four days of incubation. The importance of these four deadenylases in cell proliferation was further verified by transiently knocking down the four deadenylases in the AGS (Figure 2B) or HeLa cells (Figure 2C) using the siRNAs listed in Table S2. The dramatic inhibition of AGS and HeLa cell proliferation by depletion of the four deadenylases also suggests that their crucial role in cell proliferation was not dependent on cell types and was more likely to be a general effect.

The tumorigenicity of the MKN28 cells was studied using the nude mice model. Depletion of any one of the four deadenylases greatly inhibited tumor growth (Figure 2D and Figure S6). Among them, only very small tumors could be observed in mice with the injection of cells with the depletion of hCaf1b, while no tumors could be identified for mice injected with the hCaf1a- or hCcr4a/b-depleted cells. Taken together, these observations suggest that each deadenylase among hCcr4a/b and hCaf1a/b was indispensable for the proliferation and tumorigenesis of the MKN28 cells.

### 2.3. Depletion of hCcr4a/b or hCaf1a/b Retards Cell Cycle Progression of the MKN28 Cells

**Figure 3:**
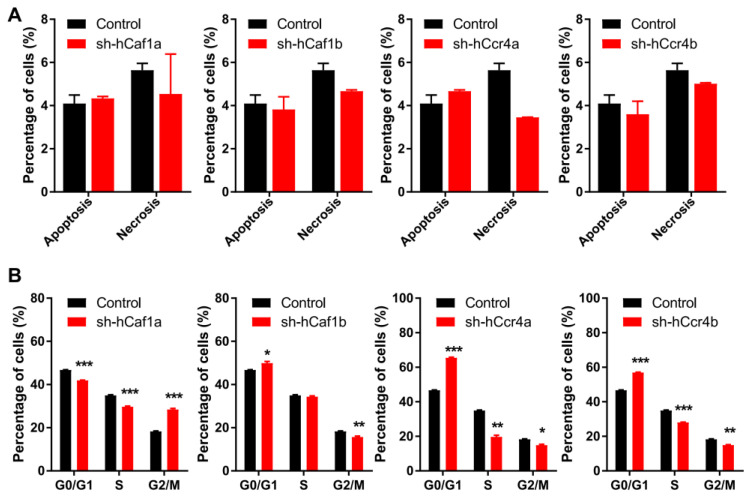
Depletion of hCcr4a/b or hCaf1a/b affects cell cycle progression but not cell death. (**A**) Percentages of apoptotic and necrotic cells analyzed by bivariate flow cytometry (*n* = 3); (**B**) Percentages of cells at the G0/G1, S, and G2/M phases analyzed by flow cytometry (*n* = 3). * *p* < 0.05, ** *p* < 0.01, and *** *p* < 0.001.

Inhibition of cell proliferation can be caused by abnormal cell cycle progression or enhanced cell death. The percentages of apoptotic and necrotic cells were obtained by bivariate flow cytometry analysis for cells stained with propidium iodide (PI) and FITC labeled annexin V (Figure S7A). As shown in Figure 3A, all of the knockdown stable cell lines had few dead cells and exhibited similar to the control, implying that depletion of hCcr4a/b or hCaf1a/b did not affect MKN28 cell death. Consistently, none of the factors promoting apoptosis including caspase 3 and PARP were activated (Figure S7B). We further analyzed cell cycle progression using flow cytometry for cells stained with PI (Figure S7C). Depletion of hCcr4a/b or hCaf1a/b resulted in dissimilar alterations in cell cycle progression (Figure 3B). The hCaf1a-depleted cells were significantly stalled at the G2/M phase, while the depletion of hCaf1b and hCcr4a/b was arrested at the G0/G1 phase to different extents. This implies that these four deadenylases might have distinct functions in cell cycle progression.

**Figure 4:**
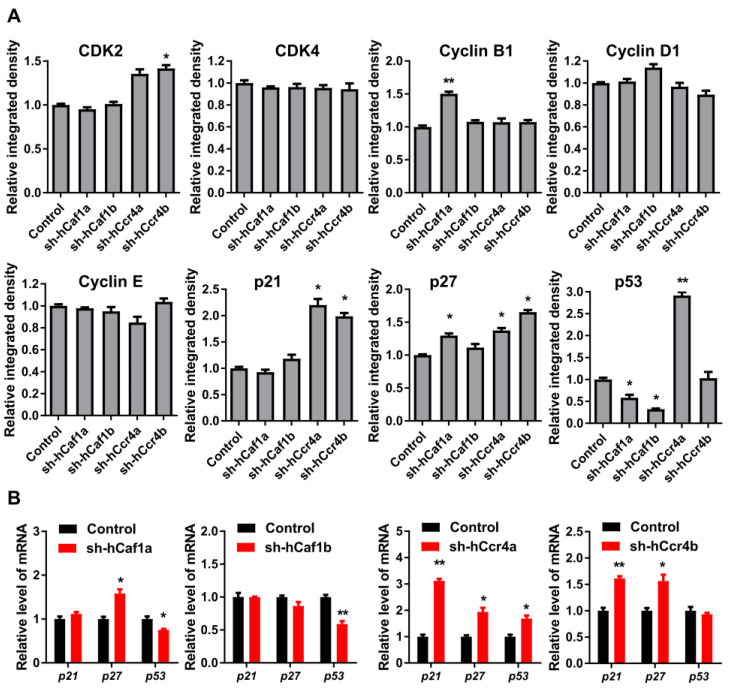
Effects of hCcr4a/b- or hCaf1a/b-knockdown on the expression of key cell cycle regulators in the MKN28 cells. (**A**) Protein levels of the cell cycle regulators determined by Western blot (*n* = 3); (**B**) The steady-state mRNA levels of *p21*, *p27,* and *p53* (*n* = 3). * *p* < 0.05, ** *p* < 0.01.

To explore the mechanism underlying cell cycle progression regulated by hCcr4a/b or hCaf1a/b, the levels of the key drivers of cell cycle progression in human cells were analyzed by Western blot (Figure 4A, Figures S8 and S11). The levels of CDKs and cyclins were not significantly altered by the depletion of one deadenylase, except for a minor increase in CDK2 and cyclin B1, suggesting that the arrest of cell cycle in the stable knockdown cells was not caused by the downregulation of cell cycle drivers. The negative regulators of the CDK–cyclin complex, p21, p27, and p53 were differentially affected by the depletion of hCcr4a/b or hCaf1a/b. The protein level of p21 was upregulated by knockdown of hCcr4a/b but not hCaf1a/b, while that of p27 was upregulated by knockdown of hCcr4a/b and hCaf1a but not hCaf1b. The amount of p53 was increased by knockdown of hCcr4a but not hCcr4b, whereas it was surprisingly decreased by hCaf1a/b. To explore whether hCaf1a/b could stabilize *p53* mRNA, the four deadenylases were overexpressed in MKN28 and HEK-293T cells and the p53 protein level was examined (Figures S9 and S11). Consistent with the decrease in p53 protein level in hCaf1a-depleted MKN28 cells, an increase was observed in the hCaf1a-overexpressing MKN28 cells. However, both knockdown and overexpression of hCcr4a induced an increase of p53 in MKN28 cells, while a decrease was observed in MKN28 cells either overexpressing or knocking down hCaf1b. Furthermore, none of the four deadenylases affected the p53 protein level in the HEK-293T cells (Figure S9B). These observations suggest that the change in p53 levels induced by hCcr4a/b or hCaf1a/b depletion was more likely to be caused by an indirect effect of the altered mRNA profile.

**Figure 5:**
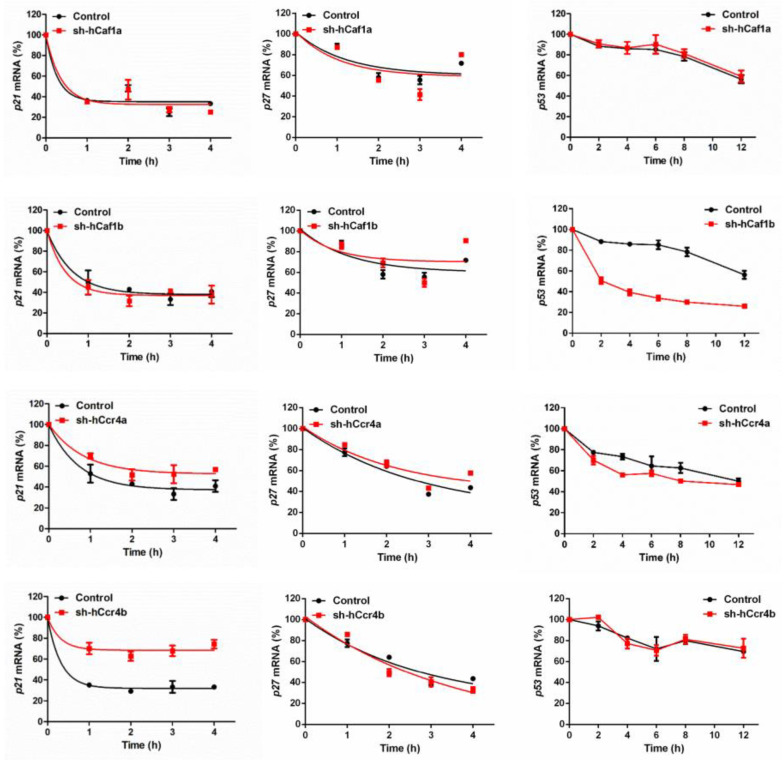
Decay kinetics of *p21*, *p27,* and *p53* mRNAs determined by quantitative real-time PCR (*n* = 3).

**Figure 6:**
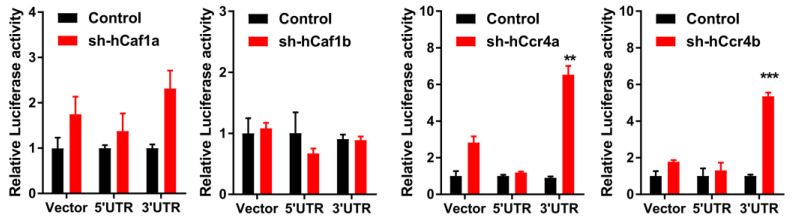
Effects of hCcr4a/b- or hCaf1a/b-knockdown on luciferase activity driven by the promoter, 5’-UTR, or 3’-UTR of *p21* in the MKN28 cells (*n* = 3). ** *p* < 0.01, and *** *p* < 0.001.

The effects of hCcr4a/b or hCaf1a/b knockdown on the steady-state mRNA levels of *p21*, *p27*, and *p53* (Figure 4B) were similar to the changes in their protein levels, suggesting that *p21*, *p27,* and *p53* mRNAs might be the targets of the hCcr4a/b and hCaf1a/b deadenylases. To verify this hypothesis, mRNA decay kinetics was determined. As shown in Figure 5, knockdown of hCcr4a/b, but not hCaf1a/b, significantly inhibited *p21* mRNA degradation, while the stability of *p27* was only moderately affected by the depletion of the four deadenylases. The decay of *p53* was greatly enhanced by hCaf1b depletion, which was consistent with the decrease that was observed for the p53 protein and mRNA levels in the hCaf1b-depleted stable cells. The almost unchanged decay kinetics in the hCaf1a- and hCcr4a-depleted cells confirmed the above deduction that the alternations in the steady-state mRNA and protein levels of p53 in these two stable cell lines (Figure 4) were caused by indirect effects of knockdown. We further confirmed the direct regulation of *p21* mRNA stability by hCcr4a/b using luciferase assay analysis of the *p21* promoter, 5′-untranslational region (UTR), or 3′-UTR (Figure 6). The unchanged luciferase activity for cells transfected with plasmids containing *p21* promoter or 5′-UTR confirmed that the change in *p21* mRNA level induced by hCcr4a/b depletion was not caused by an alteration in transcription or deadenylation-independent mRNA decay. Consistent with the results obtained from mRNA decay kinetics, an increase in the luciferase activity was only observed when plasmids containing the 3′-UTR of *p21* were transfected in cells with the depletion of hCcr4a/b but not hCaf1a/b.

### 2.4. Depletion of hCcr4a/b or hCaf1a/b Inhibits Processing Body Formation

**Figure 7:**
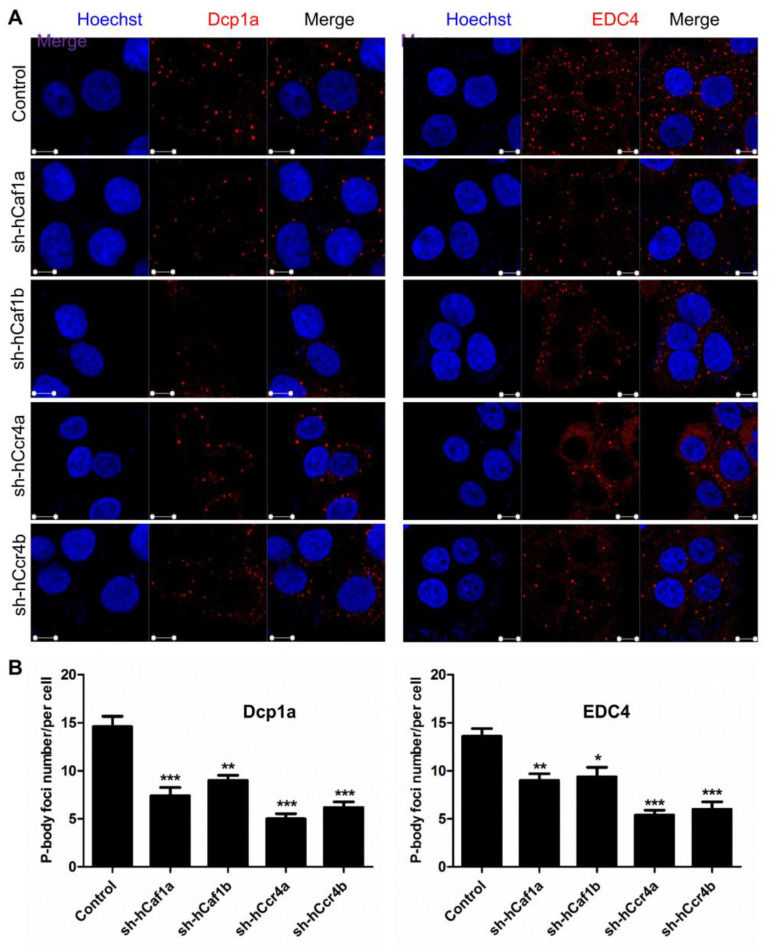
Effects of hCcr4a/b or hCaf1a/b knockdown on P-body formation in the MKN28 cells. (**A**) Representative confocal images of P-body formation visualized by two P-body marker proteins, Dcp1a and EDC4; (**B**) Quantitative analysis of the number of P-bodies per cell calculated from 10 random viewing fields for each repetition (*n* = 3). * *p* < 0.05, ** *p* < 0.01, and *** *p* < 0.001.

The above results suggested that depletion of hCcr4a/b in the MKN28 cells probably induced G0/G1 arrest by targeting *p21* mRNA, whereas the molecular mechanism underlying cell cycle arrest by hCaf1a/b knockdown remains unknown. The Ccr4–Not complex has been proposed to be the predominant cytosolic deadenylases in eukaryotic cells [12,13]. Depletion of hCcr4a/b or hCaf1a/b might affect deadenylation-related processes during mRNA turnover, and thereby the cell cycle progression might be retarded by the altered mRNA metabolism. To test this hypothesis, we determined the number of processing bodies (P-bodies) in the stable cell lines visualized by the marker proteins Dcp1a and EDC4. P-bodies have been suggested to be common cytosolic foci for the decay of untranslating mRNAs [47,48,49]. Previously, it has been shown that knockdown of PAN3, a deadenylase that shortens the long poly(A) tails of cytosolic mRNAs to medium length, affect P-body formation. Herein, we further showed that depletion of hCcr4a/b or hCaf1a/b reduced the number of P-bodies per cell by one-third to two-thirds in the MKN28 cells (Figure 7), while the effect of hCcr4a-depletion was the most significant. To further confirm the roles of the four deadenylases in P-body formation, we transiently knocked down hCcr4a/b or hCaf1a/b in the HEK-293T cells, which are a noncancer origin cell line. The P-body number per HEK-293T cell was much smaller than that per MKN28 cells (Figure S10). Nonetheless, depletion of hCcr4a/b or hCaf1a/b also gradually decreased the number of Dcp1a-positive foci per cell. Thus, our results suggest that although the two isoenzymes of hCcr4 or hCaf1 have some overlapping functions, all of them are indispensable for mRNA metabolism including P-body formation.

## 3. Discussion

It is increasingly recognized that the cancer cells reprogram multiple cellular pathways to maintain survival and continuous proliferation. The most well-known phenomenon is the reprogramed energetic metabolism that was firstly discovered by Otto Warburg in the 1920s [50]. Besides bioenergetic reprogramming, the cancer cells also need to establish new equilibrium between catabolism and anabolism including RNA metabolism. Thus, it is not surprising to find that the key enzymes and regulators involved in the RNA life cycle are dysregulated in cancer cells when compared with the normal cells. Actually, both of the two major regulators of the RNA life cycle, i.e., the RNA-binding proteins and miRNAs, have been identified to play important roles in carcinogenesis [51,52]. However, knowledge regarding the roles of ribonucleases in cancer biology is rather limited. Herein we showed that the Ccr4 and Caf1 deadenylases, which are key enzymes in cytosolic mRNA decay, were dysregulated in some types of cancers including stomach adenocarcinoma (Figure 1, Figures S1 and S2). Our results indicated that modifying the RNA decay pathway by deadenylase-depletion dramatically impaired the proliferation and tumorigenicity of gastric cancer cells MKN28 and AGS as well as the HeLa cells (Figure 2), highlighting the possibility of developing novel therapies by modifying RNA metabolism. Among the various steps of mRNA turnover, modulation of mRNA deadenylation will not affect the genome of the cells or coding sequences in the transcripts but will regulate the stability of the transcripts. Our results suggest that modulating mRNA decay is a promising point for the development of new cancer therapies. Further research is needed to verify this proposal by developing novel deadenylase inhibitors and testing their effects on tumors with high deadenylation demand.

**Figure 8:**
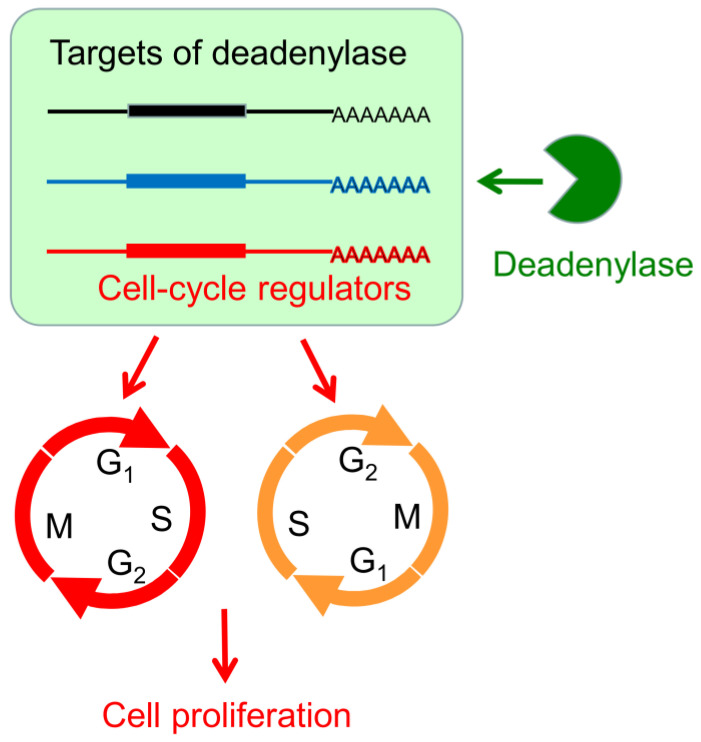
A working model demonstrating the modulation of cell proliferation by the hCaf1a/b and hCcr4a/b deadenylases. A deadenylase targets a subset of transcripts, which includes transcripts encoding cell cycle regulators. Alternations in the deadenylase level may induce G1 or G2 arrest by affecting the levels of cell cycle regulators directly or by modifying the global mRNA metabolism indirectly.

Although it is clear that deadenylase-depletion exhibited a strong antiproliferation effect on cancer cells, the underlying molecular mechanism may be very complicated involving both direct and indirect actions. In theory, deadenylase-depletion may directly alter the fate of mRNAs encoding cell cycle regulators or may indirectly affect cell cycle progression by impaired RNA turnover globally (Figure 8). Considering that p21 and p27 are the major negative regulators of the CDK–cyclin complex activity [53], upregulation of p21 and p27 probably contributes to the cell cycle arrest and retardation of cell proliferation in the hCcr4a/b-depleted cells (Figure 4). In other words, the high deadenylation activity may be important for the cancer cells with upregulated deadenylases to keep low levels of the CDK–cyclin complex inhibitors to facilitate proliferation. Previously, it has been found that depletion of Ccr4b retards NIH 3T3 cell growth by stabilizing *p27* mRNA [34]. In this research, we found that, although the steady-state p27 protein and mRNA levels were increased by hCaf1a or hCcr4a/b knockdown (Figure 4), the decay kinetics of the *p27* mRNA was not significantly altered (Figure 5). Furthermore, PARN has been shown to target *p21* mRNA in the AGS cells but not MKN28 cells [17], while herein the *p21* mRNA was identified to be the target of hCcr4a/b but not hCaf1a/b in the MKN28 cells (Figure 5 and Figure 6). The possibility that *p21* is targeted by Ccr4a/b is supported by previous observations that *p21* can be stabilized by depletion of CNOT1 in mouse embryonic fibroblasts [54] or CNOT3 in A549 lung cancer cells [21]. Both of CNOT1 and CNOT3 are the non-catalytic components of the Ccr4–Not complex, and therefore they probably regulate *p21* stability via the catalytic components Ccr4 and Caf1 isoenzymes. These observations suggest that one mRNA can be targeted by multiple deadenylases and the responsive deadenylase is strongly dependent on the cell type, probably on the different profiles of RNA-binding proteins that recruit deadenylases to the targeted mRNAs [10,14].

The mechanism underlying antiproliferation by hCaf1b depletion remains elusive since none of the tested CDK–cyclin complex inhibitors were upregulated but a dramatic decrease in the p53 protein and mRNA levels due to indirect effects (Figure 4 and Figure S9). It is worth noting that the Ccr4–Not complex has been shown to be a multifunctional platform involved in all aspects of mRNA turnover ranging from transcription to mRNA degradation [55]. Further research is needed to elucidate whether depletion of hCaf1b affects mRNA decay or other events in the MKN28 cells. Intriguingly, we observed that depletion of any one of the four deadenylases greatly impaired the formation of P-bodies in the MKN28 and HEK-293T cells (Figure 7 and Figure S10), which is consistent with the concept that deadenylation is a prerequisite to P-body formation [49]. On the contrary, depletion of PARN does not affect P-body formation in both AGS and MKN28 cells [17], suggesting that the Ccr4/Caf1 deadenylases but not PARN were responsible for the mRNA deadenylation requirement of the P-body assembly. The impaired P-body formation suggested that besides the regulation of cell cycle regulators, the Ccr4/Caf1 deadenylases had a broad impact on mRNA turnover in the MKN28 cells. The retarded mRNA metabolism might also contribute to the cell cycle arrest since it is necessary to quickly reshape the gene expression profile for phase transitions of the cell cycle progression (Figure 8).

In most studies, the actions of the Ccr4 and Caf1 deadenylases has been thought to be achieved via the Ccr4–Not complex. Moreover, although some distinct functions have been observed for the two isoenzymes of Ccr4/Caf1, it remains unclear why mammalian cells need so many deadenylases with the same catalytic functions. Herein, we found that the expression of the isoenzymes was poorly correlated (Figure S3) and they are differentially regulated in various types of tumors. This implied that the four deadenylases probably possess functions beyond the Ccr4–Not complex since the Ccr4–Not complex is comprised of one Ccr4-type and one Caf1-type deadenylases. This hypothesis was also verified by the dissimilarities in the regulation of cell cycle progression (Figure 3) and targeted mRNAs (Figure 4, Figure 5 and Figure 6). In particular, the knockdown of any one of the four deadenylases dramatically affect tumorigenicity of the MKN28 cells, suggesting that all of the four deadenylases are indispensable to MKN28 cell growth and tumorigenesis. According to these observations, we propose that the overlapping functions of the isoenzymes may be those achieved by the Ccr4–Not complex, while the distinct functions, at least part of them, may come from their separate actions.

## 4. Materials and Methods 

### 4.1. Antibodies

Rabbit anti-hCaf1a antibody was purchased from Abnova (Tokyo, Japan). Rabbit antibodies against hCaf1b, hCcr4a, Dcp1a, and EDC4 were purchased from Abcam (Cambridge, UK). Mouse anti-β-actin antibody was obtained from Sigma-Aldrich (St Louis, MO, USA). Rabbit antibodies against p21, p27, p53, cyclin D1, cyclin E2, cyclin B1, CDK2, CDK4, PARP, caspase 3, RIPK1 and hCcr4a were purchased from Cell Signaling Technology (CST) (Danvers, MA, USA). The anti-hCcr4b rabbit antibody was from OriGene (Rockville, MD, USA). The anti-hCaf1a rabbit mAb was from Proteintech (Wuhan, China), while the mouse mAb was from Bioworld (Bloomington, IN, USA). Goat anti-rabbit and mouse HRP-conjugated secondary antibodies were from Thermo Fisher Scientific (Waltham, MA, USA).

### 4.2. Clinical Samples

All experiments involving patients were approved by the Institutional Review Board of Tsinghua University (project number 20200053). Matched tumor tissues and adjacent nontumor tissues were obtained from 6 gastric cancer patients at the Department of Oncology, the first Affiliated Hospital of Zhejiang University, in September 2020. One experienced pathologist evaluated all specimens according to the World Health Organization (WHO) guidelines and the pTNM Union for International Cancer Control (UICC) pathological staging criteria. The tissues were immediately frozen in liquid nitrogen and stored at −80 °C until use. No local or systemic treatments were administered to these patients before surgery. Informed consent was obtained from all patients.

### 4.3. Cell Culture

The MKN28, AGS, HeLa, and HEK-293T cell lines were purchased from the China Center of American Type Culture Collection (ATCC, Wuhan, China) and cultured in the Dulbecco’s modified Eagle’s medium (DMEM, Gibco) with 10% fetal bovine serum (FBS, Gibco, Grand Island, NY, USA) at 37 °C with 5% CO_2_.

### 4.4. Depletion of hCaf1 and hCcr4 in the MKN28, AGS, HeLa, and HEK-293T Cells

The specific shRNA and siRNA sequences targeting hCaf1a, hCaf1b, hCcr4a, and hCcr4b was synthesized in Sangon Biotech (Shanghai, China). The shRNA sequences targeting hCaf1a, hCaf1b, hCcr4a, and hCcr4b are provided in Table S1, while the siRNA sequences are listed in Table S2.

The shRNA oligonucleotides were cloned into the pSilencer 2.1-U6 hygro vector (Ambion, Waltham, MA, USA). The negative control vector was supplied within the kit (Ambion, Waltham, MA, USA) and the sequence was 5′-GATCCACTACCGTTGTTATAGGTGTTCAAGAGACACCTATAACAACGGTAGTTTTTTGGA-3′. The MKN28 cells were seeded into 6-well plates at 60–70% confluence before transfection, and then transfected with 1 μg vector using Lipofectamine^®^ 3000 reagent (Invitrogen, Waltham, MA, USA), according to the manufacturer’s instruction. The transfected MKN28 cells were maintained in selective DMEM medium containing hygromycin B (Invitrogen, Waltham, MA, USA) with a concentration of 200 μg/mL. The stable hCaf1a-, hCaf1b-, hCcr4a- and hCcr4b-knockdown cell lines were obtained after three weeks of selection. The knockdown efficiencies of hCaf1a, hCaf1b, hCcr4a, and hCcr4b were confirmed by qRT-PCR and Western blot analysis.

Transient knockdowns of the four deadenylases in the AGS, HeLa, and HEK-293T cells were achieved by seeding the cells into 12-well plates at 30–40% confluence before transient transfection, and then transfected with 100 nM siRNA using 2 μL INTERFERin regeant (Polyplus Transfection, New York, NY, USA), according to the manufacturer’s instruction. After 24 h, a second round of knockdown was performed by transfecting with 1.5 μg siRNA using Lipofectamine^®^ 3000 reagent (Invitrogen, Waltham, MA, USA), according to the manufacturer’s instruction. The knockdown efficiencies of hCaf1a, hCaf1b, hCcr4a, and hCcr4b were determined by qRT-PCR and Western blot analysis.

### 4.5. Quantitative Real-Time PCR (qPCR)

Total RNA was extracted from the cells using a TIANGEN RNAprep Pure Cell Kit (Beijing, China) or from gastric tissue powders using the TRIzol reagent (Invitrogen, Carlsbad, CA, USA), according to the manufacturer’s protocols. Reverse transcription was performed with a PrimeScript^TM^RT Master Mix (Takara, Beijing, China) using 1 μg total RNA. The qPCR reactions were run with EvaGreen Dye (Biotium, Fremont, CA, USA) or Hieff qPCR SYBR Green Master Mix (Yeasen, Shanghai, China) in triplicate on a MX3005P cycler (Agilent, Santa Clara, CA, USA). The specific primers used for qPCR were the same as those described previously [17]. Relative mRNA levels were determined using the comparative Ct method and normalized by the *GAPDH* mRNA level.

### 4.6. Western Blotting

Cancer tissues were fast frozen in liquid nitrogen and pulverized. The tissue powders and cultured cells were lysed with radioimmuno-precipitation assay (RIPA) lysis buffer (Beyotime Biotechnology, Shanghai, China) supplemented with 10 μL/mL phenylmethanesulfonyl fluoride (PMSF) (Biodee Biotechnology, Beijing, China). Protein concentrations were determined by a Bicinchoninic Acid (BCA) Protein assay kit (Solarbio Science & Technology, Beijing, China). Equal amounts of protein were separated by SDS-PAGE, and then transferred to PVDF membranes (Millipore, Billerica, MA, USA). Then, the membranes were blocked with 5% non-fat milk at room temperature for 2 h before being incubated with the appropriate primary antibodies at 4 °C overnight. The membranes were subsequently incubated with the appropriate HRP-conjugated secondary antibodies for 1 h at room temperature, then, visualized by an Enhanced Chemiluminescence Detection kit (Thermo Scientific, Waltham, MA, USA) and photographed by a Bio-Rad Chemidoc MP imaging system. The primary antibodies used in this research included rabbit anti-hCaf1a, -hCaf1b, -hCcr4a, -hCcr4b, -p21, -p27, -p53, -cyclin D1, -cyclin E2, -cyclin B1, -CDK2, -CDK4, -PARP, -RIPK1 and -caspase-3 antibodies (1:400 to 1:1000) and the internal controls anti-β-actin and -GAPDH antibodies (1:10,000).

### 4.7. Cell Proliferation Assay

Cell proliferation was determined by CellTiter 96 Aqueous One Solution Proliferation Assay Kit (Promega, Madison, WI, USA). The cells were seeded into the 96-well culture plates with a density of 2.5 × 10^3^ cells/well. After incubation for a given time, 10 μL of MTS solution was added to the each well with 100 μL fresh medium and the cells were cultured for another 1 h. Cell proliferation ability was determined by measuring the absorbance at 490 nm (OD_490nm_) using a microplate reader (Bio-Rad, Hercules, CA, USA). The experimental time intervals were 0, 1, 2, 3, 4, and 5 days for the MKN28 and AGS cells, 0, 0.5, 1, 1.5, 2 and 2.5 days for the HeLa cells.

### 4.8. Cell Apoptosis Assay

The harvested cells were stained with Annexin V-FITC/PI apoptosis detection kit (BD Biosciences, San Jose, CA, USA), according to the manufacturer’s instructions. Cell apoptosis analysis was carried out on a FACSCalibur flow cytometer (BD Biosciences, San Jose, CA, USA).

### 4.9. Cell Cycle Analysis

The harvested cells were fixed with 70% cold ethanol at 4 °C overnight. After washing with PBS twice, cells were incubated with 100 μg/mL RNase A, in PBS, at 37 °C for 30 min, and then stained with 50 μg/mL propidium iodide (PI) for 30 min in the dark. Cell cycle analysis was detected by FACSCalibur flow cytometer (BD Biosciences, San Jose, CA, USA). The percentage of cell populations in the G_0_/G_1_, S, and G_2_/M phases of the cell cycle was analyzed with ModFit LT 3.0 software.

### 4.10. Immunofluorescence Analysis

Cells were grown on coverslips preplaced in the 6-well culture plates. After 24 h cultivation, cells were fixed with 4% paraformaldehyde, and then permeabilizated with 0.2% Triton X-100 in PBS for 30 min. Then, the cells were blocked with 10% goat serum for 1 h at room temperature. Immunofluorescence staining was determined by Dcp1a and EDC4 primary antibodies at 4 °C overnight. After washing three times with PBS, cells were incubated with the Cy5-conjugated anti-rabbit secondary antibody (Jackson ImmunoResearch Laboratories, West Grove, PA, USA) for 1 h at room temperature. The nuclei of cells were counterstained with Hoechst 33,342 (Invitrogen, Carlsbad, CA, USA) for 1 min and washed with PBS. The stained cells were mounted using Fluoromount-G (Southern Biotechnology Associates, Birmingham, AL, USA), and subsequently observed using a Carl Zeiss LSM 710 confocal microscope system.

### 4.11. Determination of mRNA Stability

Cells were cultured in 12-well plates prior to transcription inhibition treatment with 5 μg/mL actinomycin D (Sigma-Aldrich, St Louis, MO, USA) for 0, 1, 2, 3, 4, 6, 8, 10, or 12 h. After treatment at different time points, the cells were collected and the total RNAs were extracted by standard procedures. The abundance of *p21*, *p27,* or *p53* mRNA was measured by RT-qPCR and normalized by an internal control of *GAPDH* mRNA. The results were normalized by the untreated sample and presented by fitting the time-course data using the first-order exponential decay kinetics.

### 4.12. Dual Luciferase Reporter Assay

Cells were seeded in 24-well plates and transfected with p21 5′-UTR, 3′-UTR, and control luciferase reporter constructs; pRL-TK was also co-transfected and used as an internal control. After transfection for 24 h, the luciferase activities were measured using the Dual-Luciferase Reporter Assay kit (Vigorous, Beijing, China), according to the manufacturer’s instructions. The firefly luciferase reporter activity of each sample was normalized to the control Renilla activity.

### 4.13. In Silicon Analysis

Gene Expression Profiling Interactive Analysis [46] (GEPIA, http://gepia.cancer-pku.cn/index.html, accessed date: 9 November 2020), an interactive web server, was used for the analysis of the TCGA and GTEx data. The RNA-seq expression data of *hCaf1a*, *hCaf1b*, *hCcr4a,* and *hCcr4b* in TCGA tumor groups and TCGA and GTEx normal tissue groups were compared and analyzed by the standard parameters provided by GEPIA.

### 4.14. Tumorigenicity in Nude Mice

The female athymic BALB/c nude mice aged 4 weeks were randomly divided into 5 groups and maintained under specific pathogen-free conditions. Then, 5 × 10^6^ MNK28 stable cells with sh-hCcr4a, sh-hCcr4b, sh-hCaf1a, sh-hCaf1b, or pSilencer hygro vector were resuspended in 200 μL 1:1 (v/v) Matrigel (Corning, NY, USA) and PBS mixture, and subcutaneously injected into the left sub-axillary of each mouse. The tumor sizes were measured with a Vernier caliper (ASONE, Japan) and recorded every other day. The tumor volume was calculated using the formula of tumor volume (mm)^3^ = (length (mm)) (width (mm))^2^ × 0.5. After the inoculation of tumors for 2 months, photos of mice and excised xenografted tumors were taken. Animal experiments and procedures were conducted in accordance with the guidelines and approval of the Institutional Animal Care and Use Committee, Tsinghua University (Assurance Identification Number/Ethical Protocol Code 18-YYB1).

### 4.15. Statistical Analysis

Most experiments were repeated with at least three biological replicates, which were done using different sets of cells or tissues. The significance of data between two independent groups was analyzed using a two-tailed Student’s *t*-test or two-way ANOVA test using GraphPad Prism 7.04 (GraphPad Software Inc., La Jolla, CA, USA). All the data were presented as mean ± SEM. A *p* value smaller than 0.05 was considered to be statistically significant.

## 5. Conclusions

By regulating RNA fate, deadenylases have been found to participate in diverse cellular processes including cell proliferation, DNA damage response, and genome stability, which are closely linked to carcinogenesis. However, the roles of various deadenylases in carcinogenesis are rarely addressed in the literature. In this study, we found that Ccr4 and Caf1, the major cytosolic deadenylases in eukaryotic cells, are dysregulated in several types of cancers including stomach adenocarcinoma. Depletion of hCaf1a/b or hCcr4a/b dramatically inhibited proliferation of the MKN28, AGS, and HeLa cells. Mechanistic studies indicated that the depletion of hCaf1a/b or hCcr4a/b resulted in dissimilar patterns of cell cycle arrest but did not significantly affect cell death. Upregulation of p21 and p27 might contribute to the cell cycle arrest in the MKN28 cells with the depletion of hCaf1a or hCcr4a/b. We identified that the *p21* mRNA was a target of hCcr4a/b but not of hCaf1a/b. Depletion of the hCcr4a/b or hCaf1a/b deadenylase significantly impaired P-body formation in the MKN28 or HEK-293T cells, implying that RNA metabolism was globally affected by the depletion of hCcr4a/b or hCaf1a/b. More importantly, depletion of any one of the four deadenylases dramatically reduced proliferation and tumorigenicity of the MKN28 cells. Our results highlight that the modulation of deadenylase activity might be a potential way for the development of cancer treatment strategies.

## Data Availability

The data presented in this study are available in the article and supplementary material.

## Supplementary Materials

The following are available online at https://www.mdpi.com/2072-6694/13/4/834/s1, Figure S1: The deadenylases hCaf1a, hCaf1b, hCcr4a, and hCcr4b were dysregulated in several type of cancers analyzed by the web server GEPIA, Figure S2: Correlation analysis of gene expression between hCaf1a/b and hCcr4a/b, Figure S3: Representative Western blot analysis of the protein levels of hCaf1a/b and hCcr4a/b in patients samples, Figure S4: Knockdown efficiency of various shRNAs, Figure S5: Knockdown efficiency of the four deadenylases in MKN28, AGS, and HeLa cell lines, Figure S6: Photos of nude mice after the inoculation of tumors for 2 months, Figure S7: Depletion of hCaf1a/b or hCcr4a/b impaired cell cycle progression but not cell death, Figure S8: Representative Western blot analysis of various drivers and regulators of cell cycle progression, Figure S9: Effect of hCaf1a/b or hCcr4a/b overexpression on the p53 level in MKN28 or HEK-293T cells, Figure S10: Effect of hCcr4a/b or hCaf1a/b knockdown on P-body formation in the HEK-293T cells, Figure S11: Original Western blot gels, Table S1: The shRNA sequences targeting hCaf1a, hCaf1b, hCcr4a, and hCcr4b to generate stable knocking down cell lines, Table S2: The siRNA sequences targeting hCaf1a, hCaf1b, hCcr4a, and hCcr4b.
