# Supplementary material for: Human Ccr4 and Caf1 Deadenylases Regulate Proliferation and Tumorigenicity of Human Gastric Cancer Cells via Modulating Cell Cycle Progression
Source: Cancers (Basel). 2021 Feb 17;13(4):834. doi: 10.3390/cancers13040834 (PMC7922635; doi:10.3390/cancers13040834)
Supplement: Supplementary file 1 [file cancers-13-00834-s001.pdf]

Supplementary Materials: Human Ccr4 and Caf1 deadenylases regulate proliferation and tumorigenicity of human gastric cancer cells via modulating cell cycle progression

Xiao-Hui Song, Xiao-Yan Liao, Xu-Ying Zheng, Jia-Qian Liu, Zhe-Wei Zhang, Li-Na Zhang and Yong-Bin Yan

A: *hcaf1a*

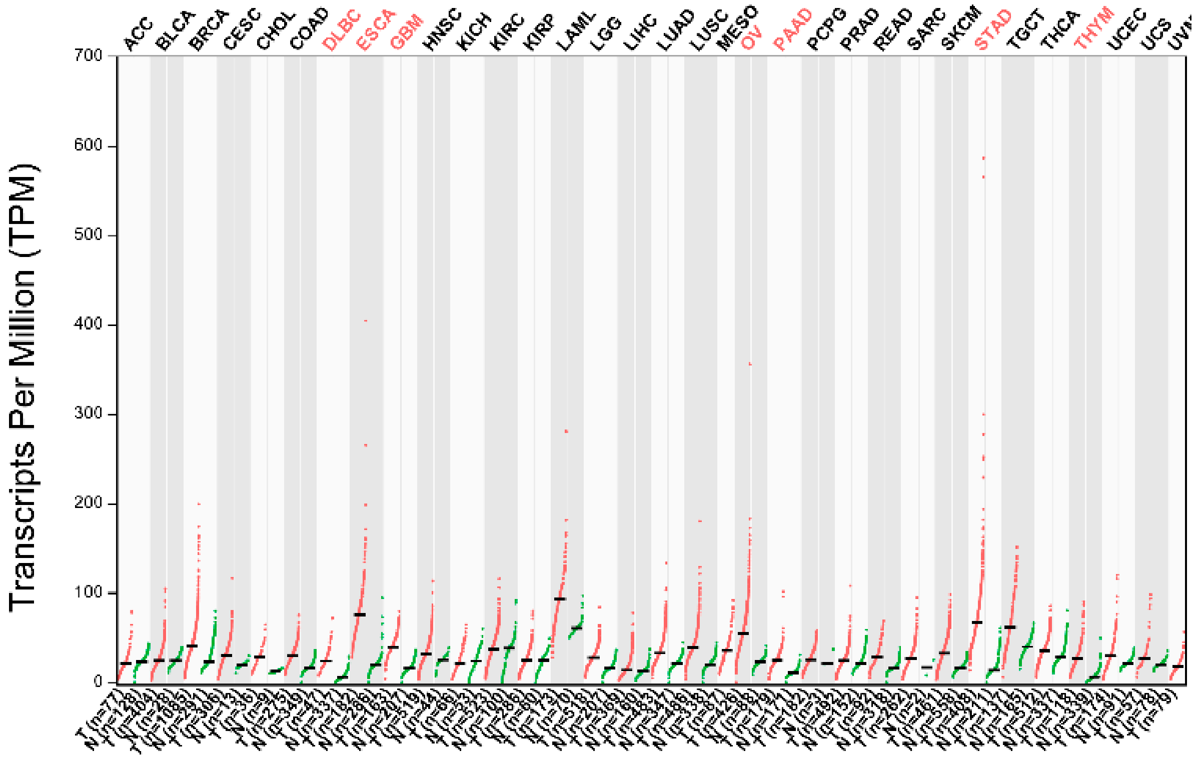

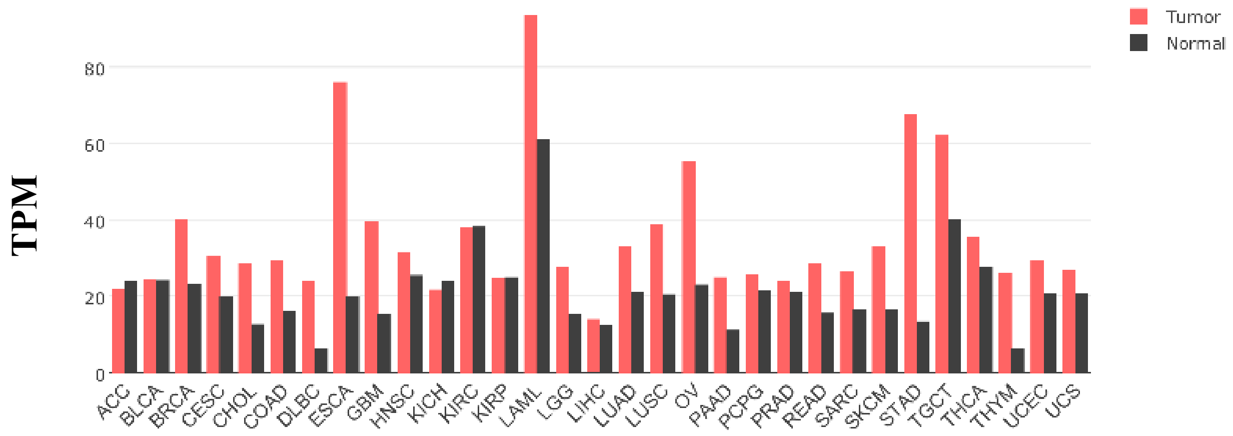

B: *hcaf1b*

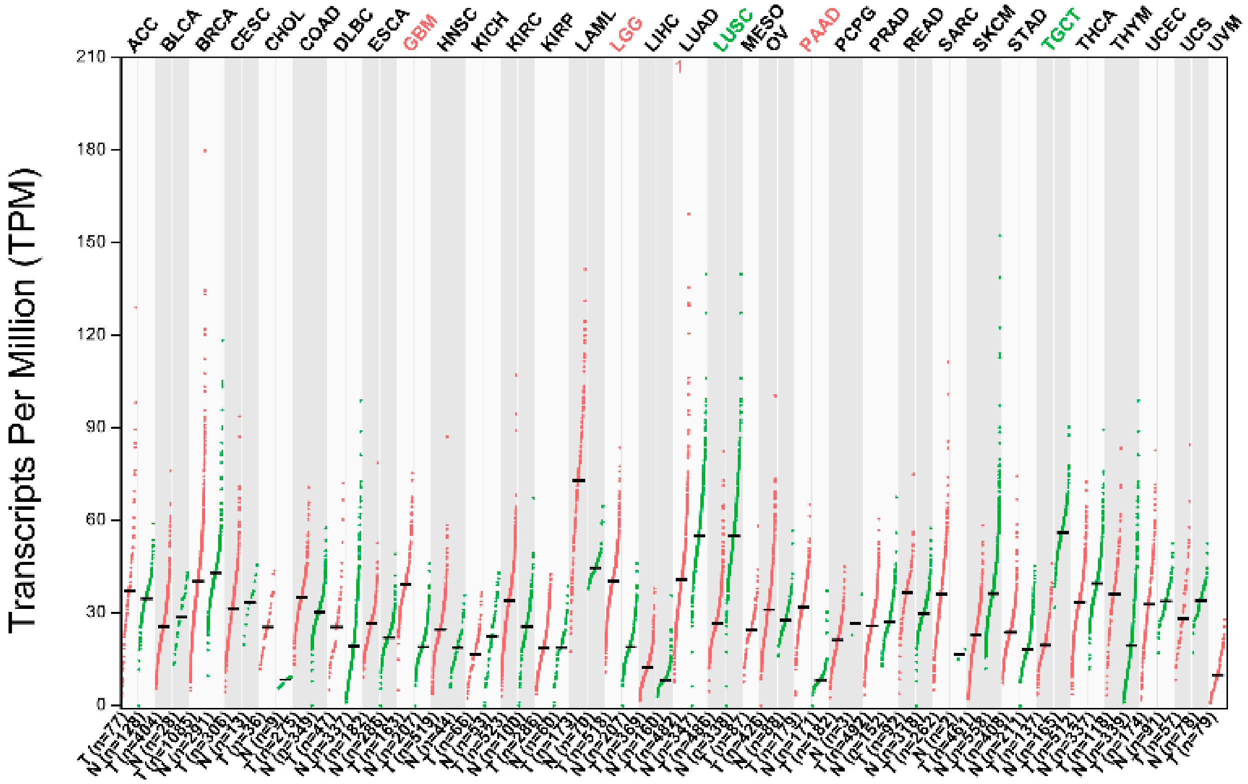
ACC
BLCA
BRCA
CESC
CHOL
COAD
DLBC
ESCA
GBM
HNSC
KICH
KIRC
KIRP
LAML
LGG
LIHC
LUAD
LUSC
MESO
OV
PAAD
PCPG
PRAD
READ
SARC
SKCM
STAD
TGCT
THCA
THYM
UCEC
UCS
UVM
Transcripts Per Million (TPM)
0
30
60
90
120
150
180
210

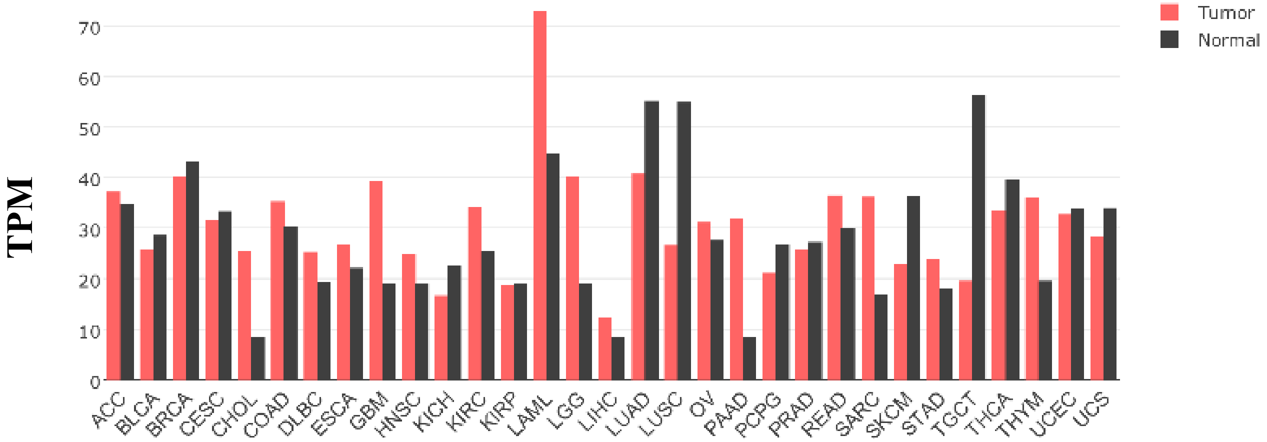
TPM
0
10
20
30
40
50
60
70
Tumor
Normal
ACC
BLCA
BRCA
CESC
CHOL
COAD
DLBC
ESCA
GBM
HNSC
KICH
KIRC
KIRP
LAML
LGG
LIHC
LUAD
LUSC
OV
PAAD
PCPG
PRAD
READ
SARC
SKCM
STAD
TGCT
THCA
THYM
UCEC
UCS

C: *hccr4a*

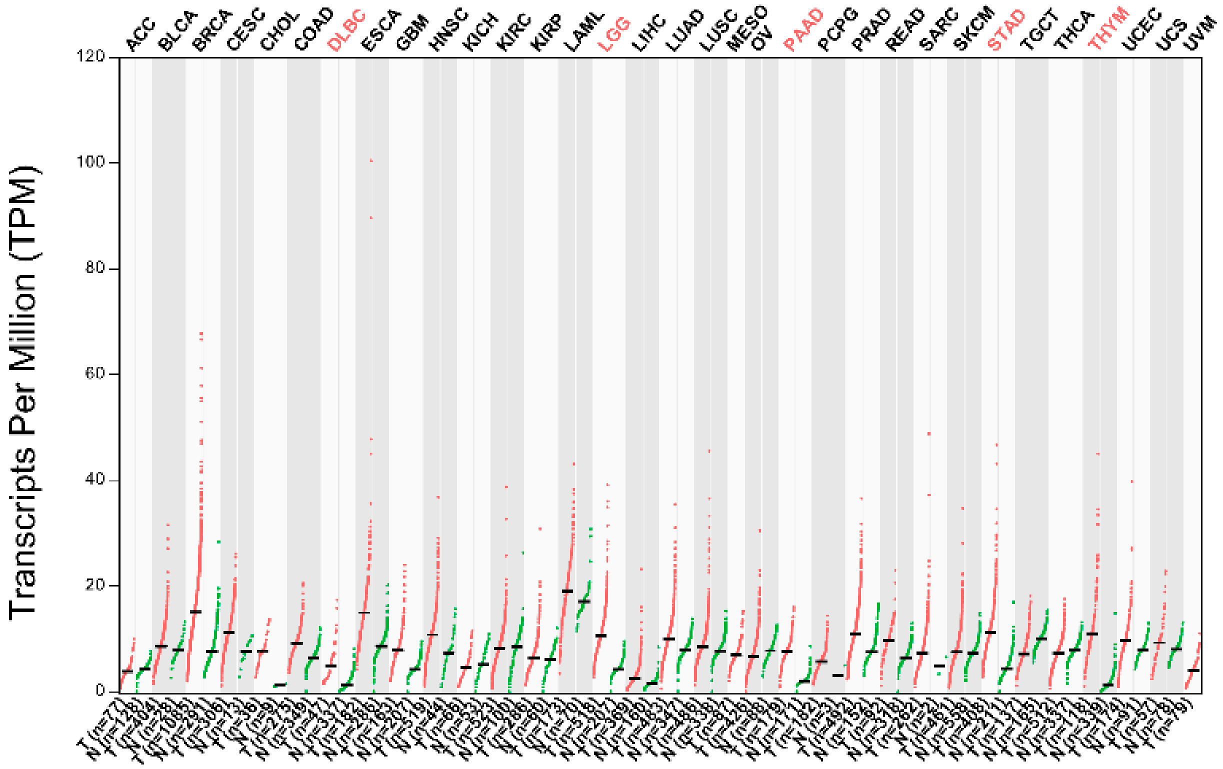
Transcripts Per Million (TPM)
ACC
BLCA
BRCA
CESC
CHOL
COAD
DLBC
ESCA
GBM
HNSC
KICH
KIRC
KIRP
LAML
LGG
LIHC
LUAD
LUSC
MESO
OV
PAAD
PCPG
PRAD
READ
SARC
SKCM
STAD
TGCT
THCA
THYM
UCEC
UCS
UVM
0
20
40
60
80
100
120

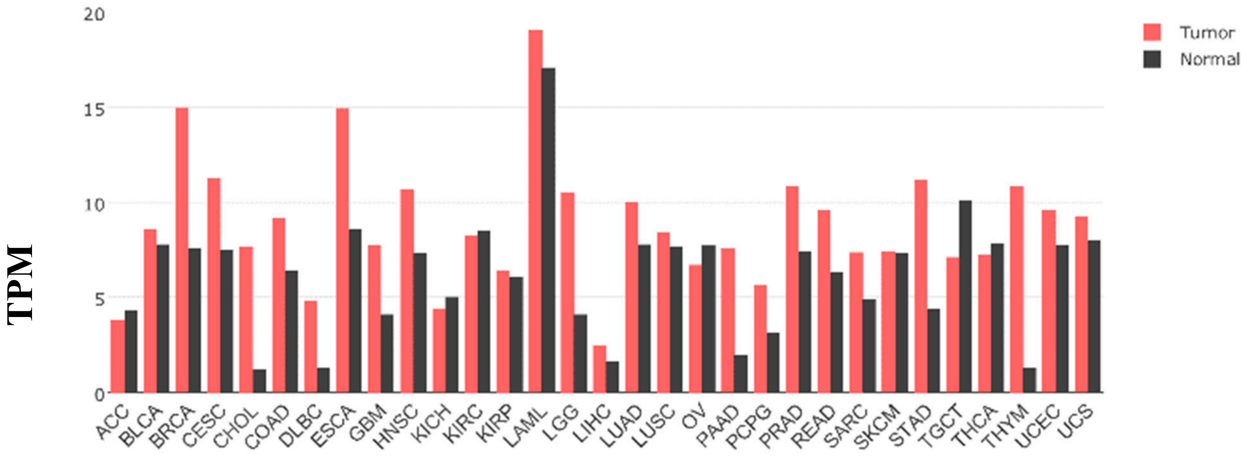
TPM
Tumor
Normal
0
5
10
15
20
ACC
BLCA
BRCA
CESC
CHOL
COAD
DLBC
ESCA
GBM
HNSC
KICH
KIRC
KIRP
LAML
LGG
LIHC
LUAD
LUSC
OV
PAAD
PCPG
PRAD
READ
SARC
SKCM
STAD
TGCT
THCA
THYM
UCEC
UCS

D: *hccr4b*

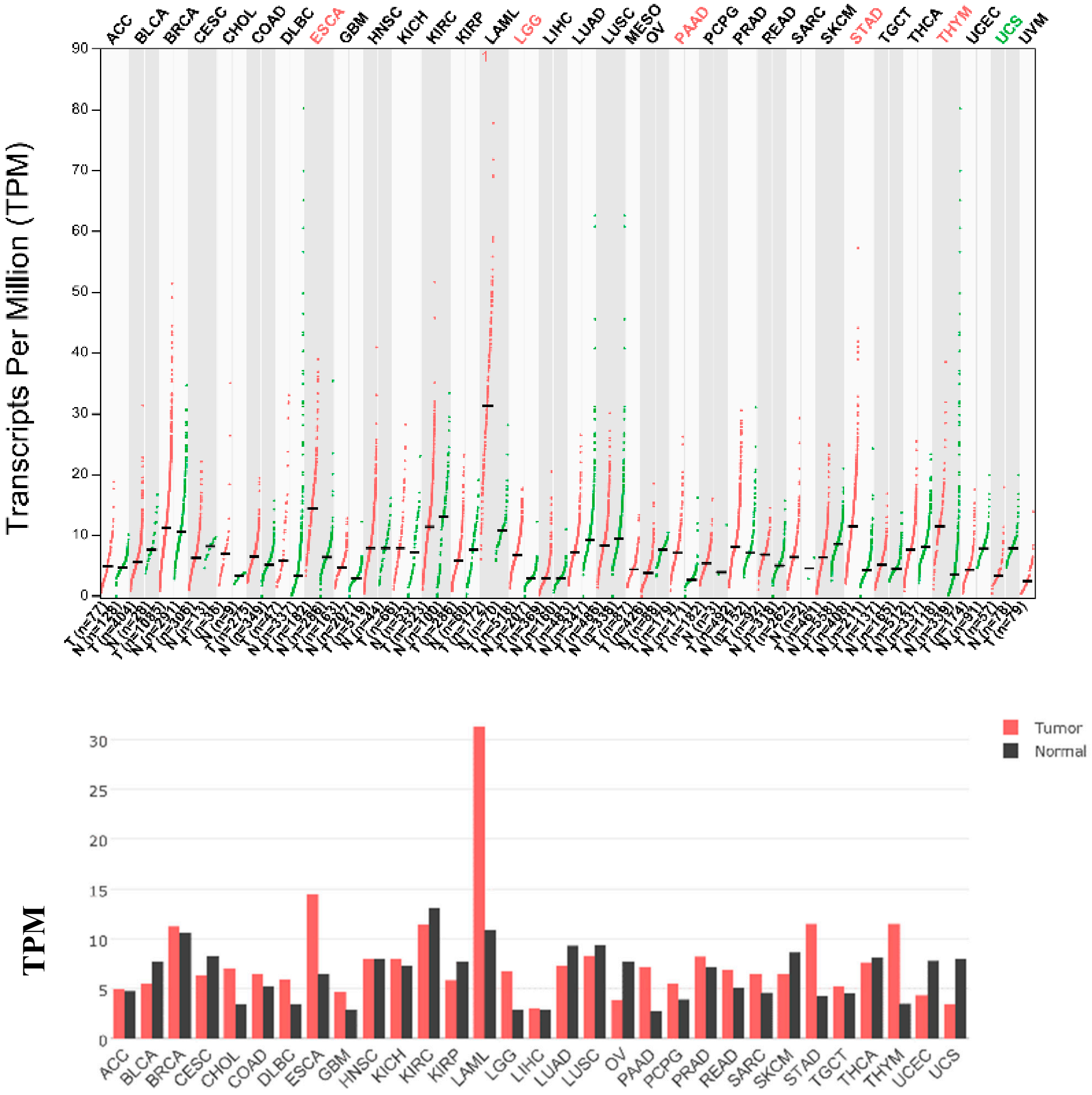

Figure S1. The deadenylases hCaf1a (**A**), hCaf1b (**B**), hCcr4a (**C**) and hCcr4b (**D**) were dysregulated in several type of cancers analyzed by the web server GEPIA (http://gepia.cancer-pku.cn/index.html). The names of various types of tumors follow the TCGA abbreviations. The tumor names with upregulated genes were highlighted in red, while those with downregulated genes were shown in green ($p < 0.01$). In each panel, the upper and the bottom ones show the dot plot and bar blot of gene expression profile across all tumor samples and paired normal tissues, respectively.

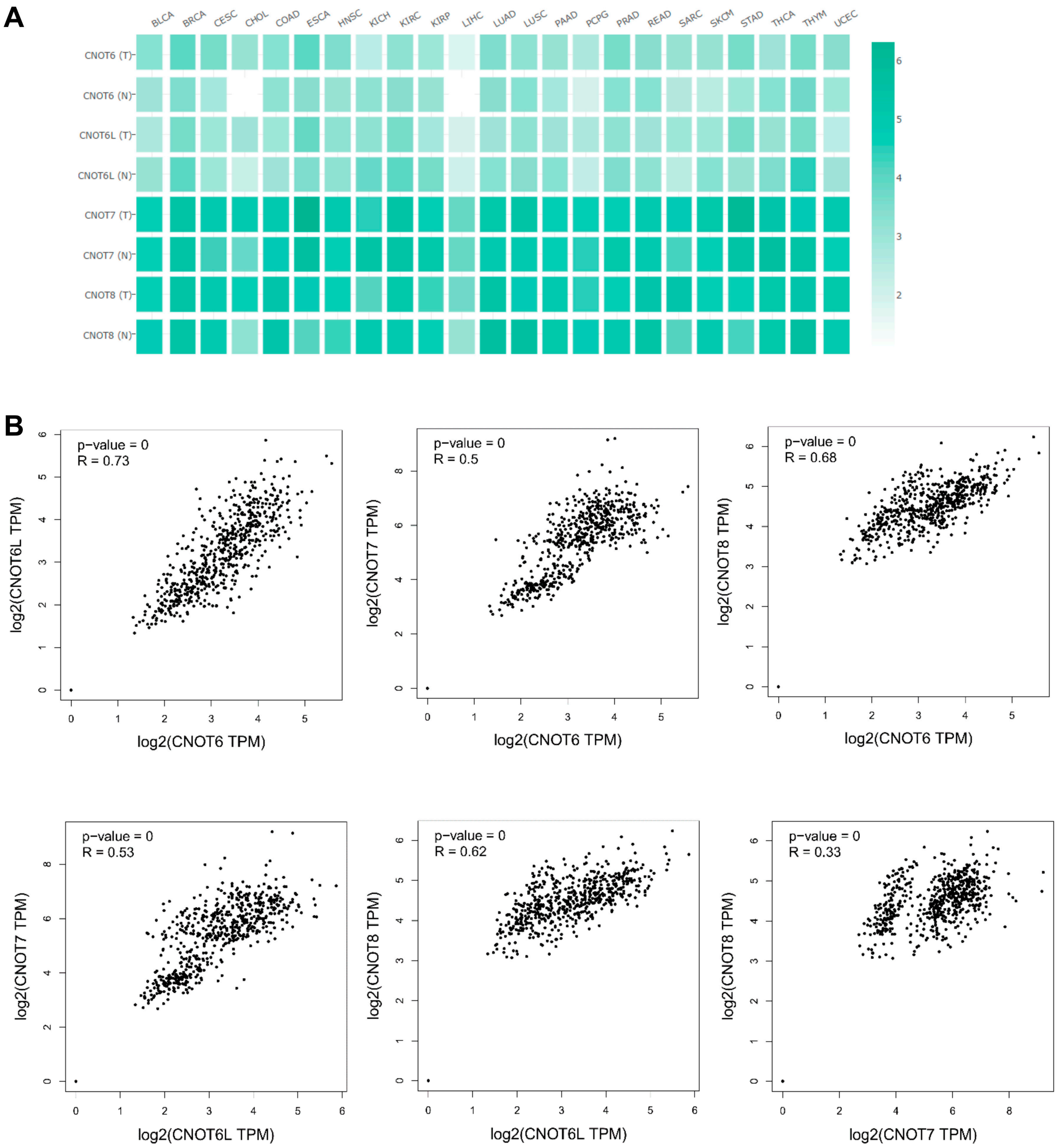

Figure S2. Gene expression comparison and correlation analysis between hCaf1a/b and hCcr4a/b. (**A**) Gene expression matrix plots of hCaf1a/b and hCcr4a/b in various types of tumors as well as the paired normal tissues. The values are calculated by log_2(TPM+1), and TPM stands for transcripts per million. (**B**) Pair-wise gene expression correlation analysis. R value is the Pearson correlation coefficient. The analysis was performed using the online web server GEPIA (http://gepia.cancer-pku.cn/index.html).

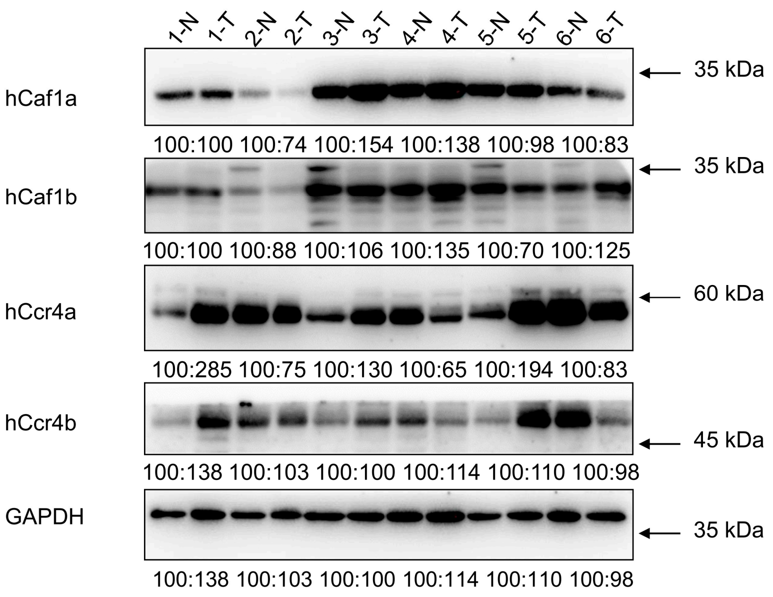

Figure S3. Representative western blot analysis of the protein levels of hCaf1a/b and hCcr4a/b in patients samples. N denotes normal tissues, while T represents tumors.

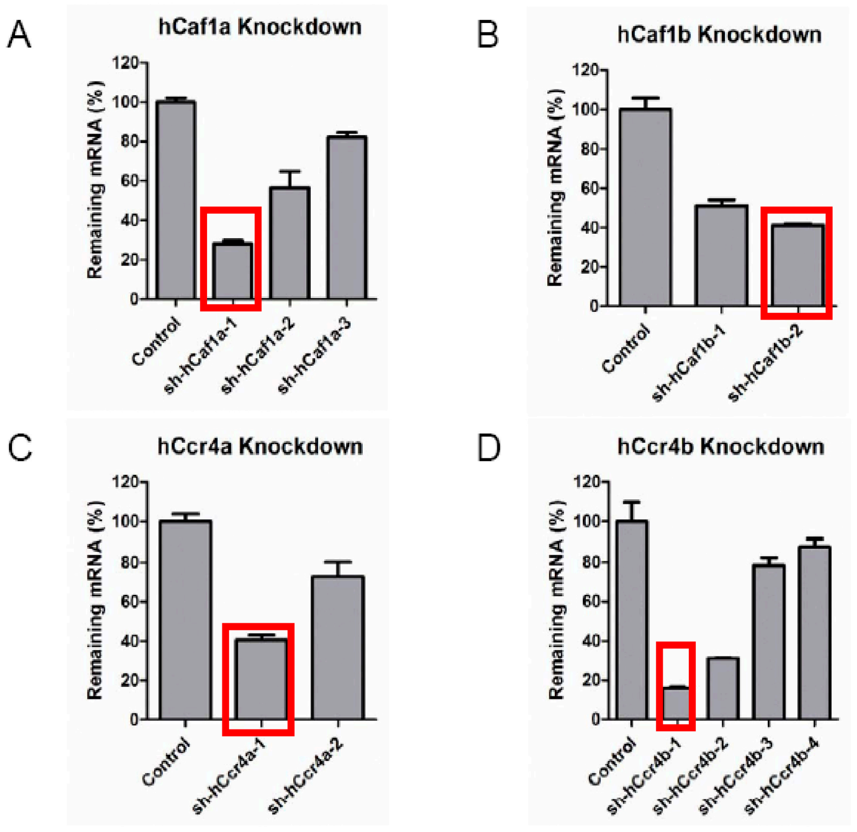

Figure S4. Knockdown efficiency of various shRNAs. Those highlighted with red box were used for further experiments.

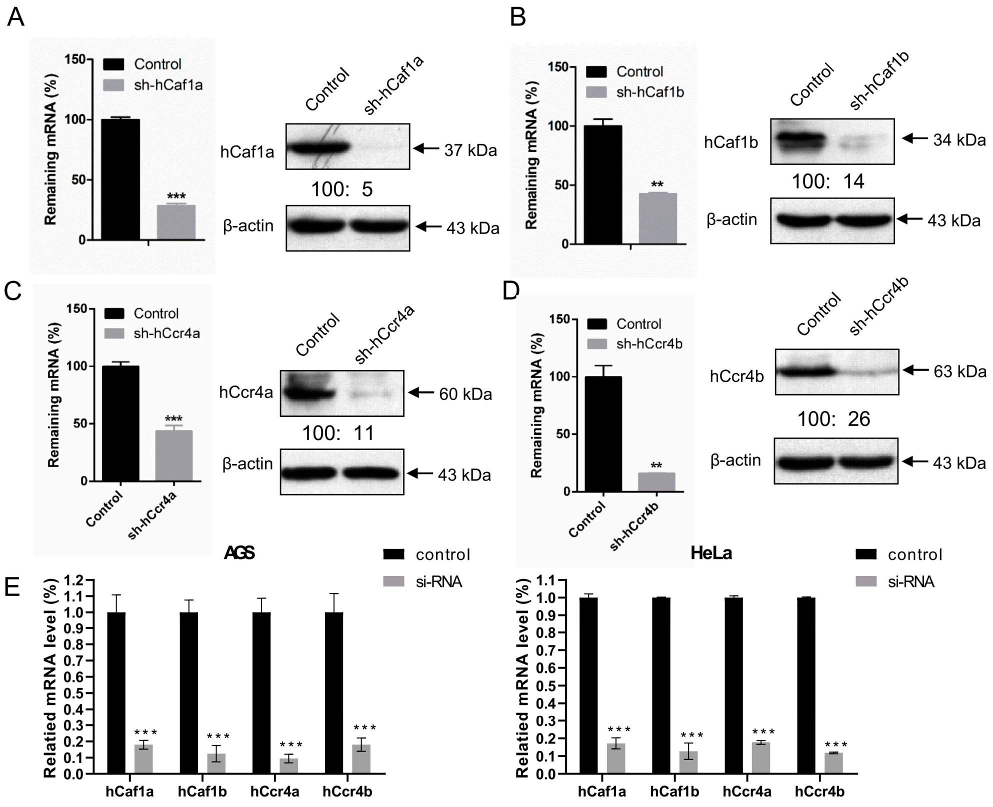

Figure S5. Knockdown efficiency of the four deadenylases in MKN28, AGS and HeLa cell lines: (**A**) MKN28 cells with sh-hCaf1a; (**B**) MKN28 cells with sh-hCaf1b; (**C**) MKN28 cells with sh-hCcr4a; (**D**) MKN28 cells with sh-hCcr4b; (**E**) AGS cells with transient knockdown of the four deadenylases by siRNA; (**F**) HeLa cells with transient knockdown of the four deadenylases by siRNA. The MKN28 stable cell lines were established using shRNAs, while the transiently knockdown AGS and HeLa cells were obtained by transient transfection of siRNAs

Control sh-hCaf1b sh-hCaf1a sh-hCcr4b sh-hCcr4a

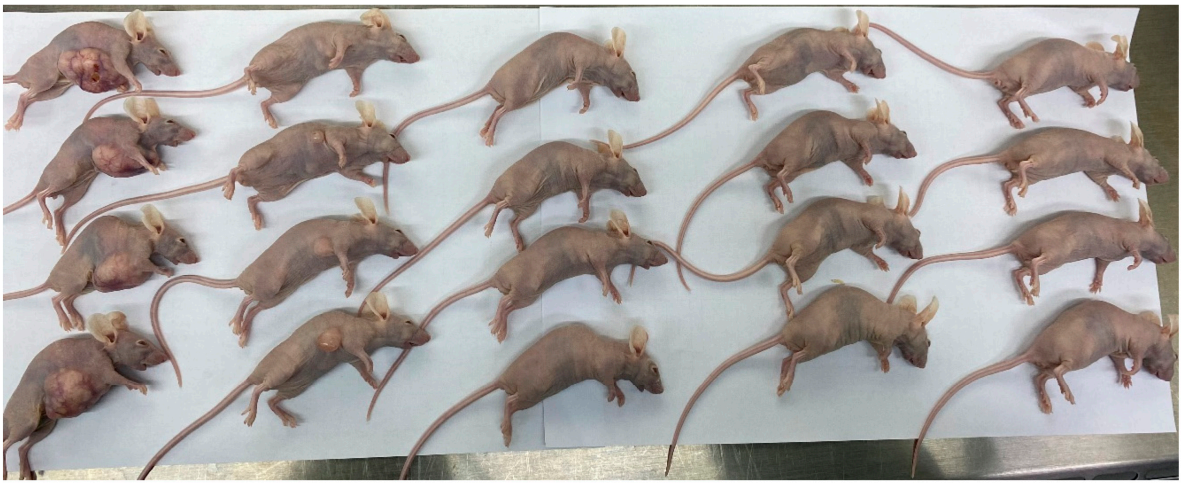

Figure S6. Photos of nude mice after the inoculation of tumors for 2 months.

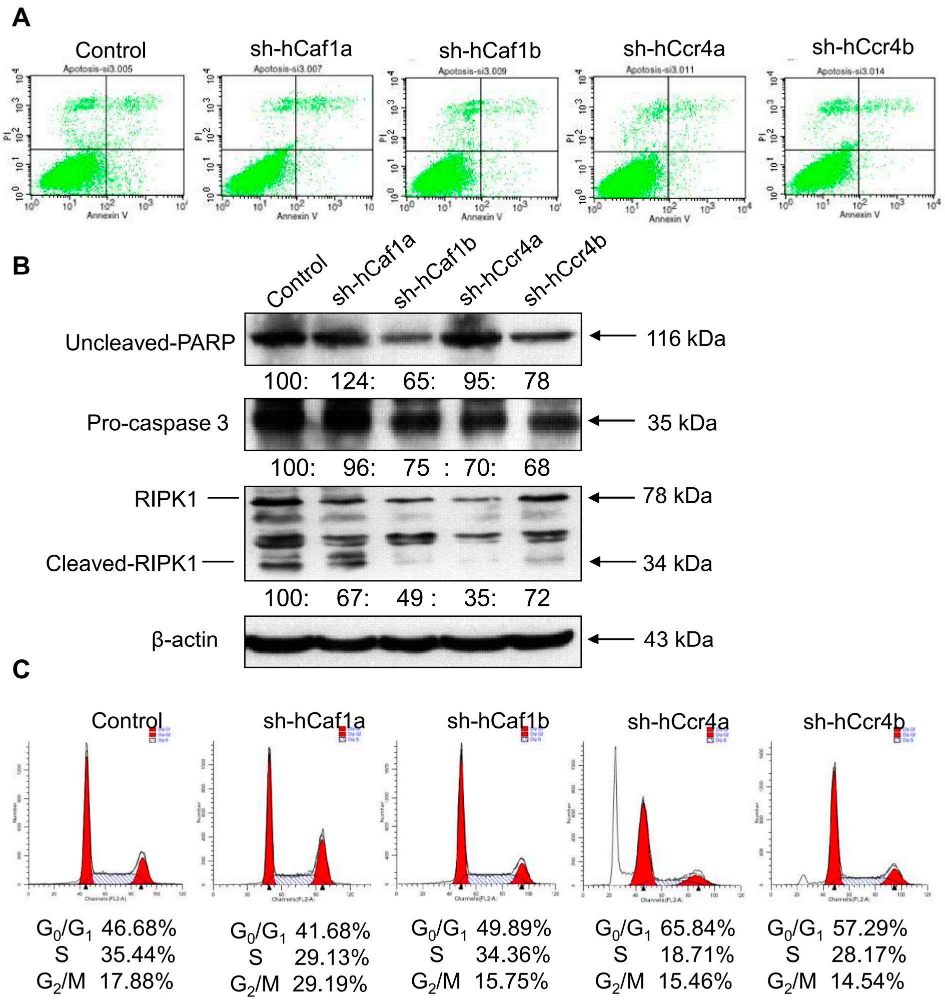

Figure S7. Depletion of hCaf1a/b or hCcr4a/b impaired cell cycle progression but not cell death. (**A**) Representative bivariate flow cytometry analysis of cell death. (**B**) Western blot analysis of marker proteins involved in apoptosis. (**C**) Representative flow cytometry analysis of cell cycle progression.

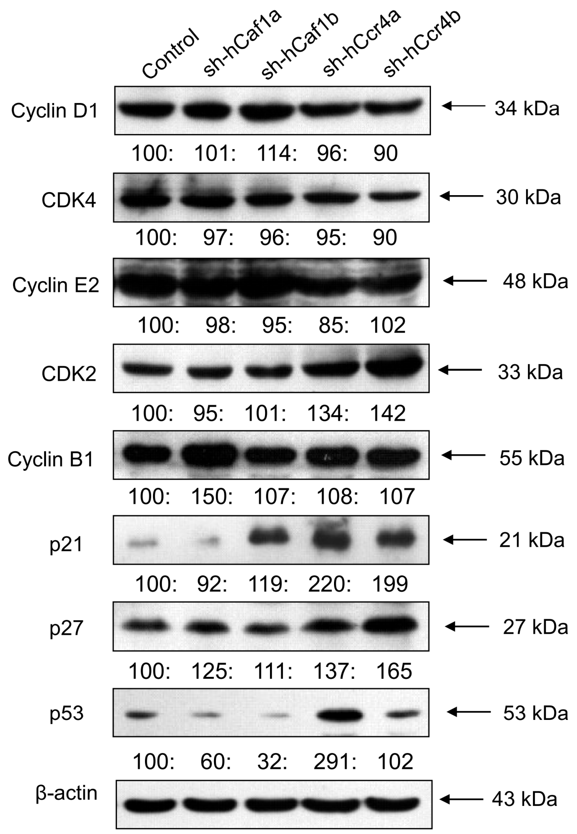

Figure S8. Representative western blot analysis of various drivers and regulators of cell cycle progression.

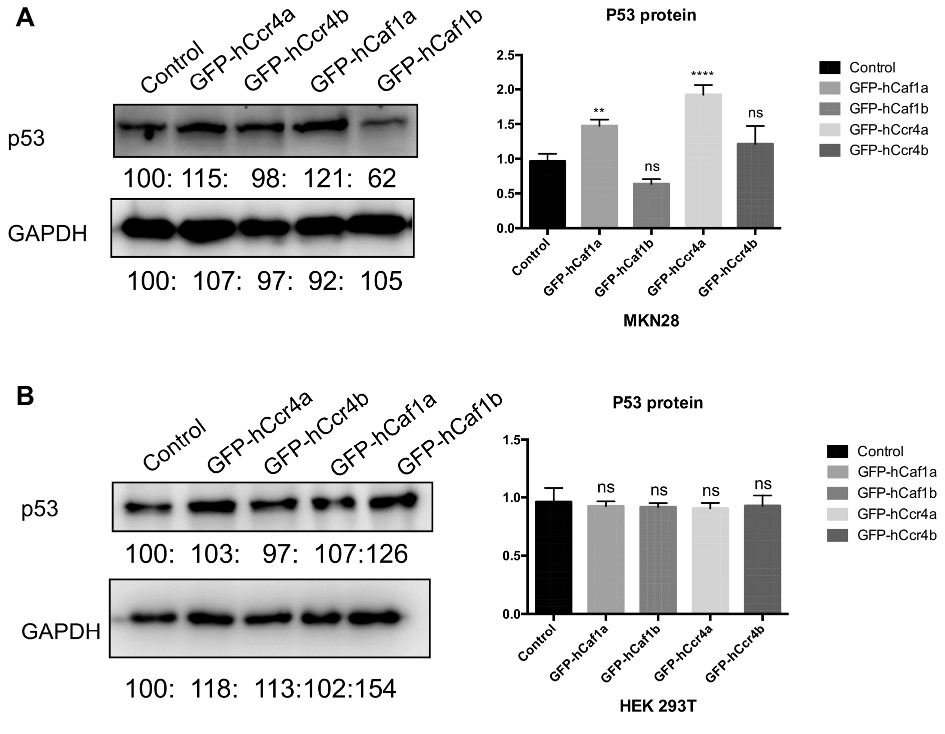

Figure S9. Effect of hCaf1a/b or hCcr4a/b overexpression on the p53 level in MKN28 (**A**) or HEK-293T cells (**B**). Left panel, representative western blot analysis; right panel, quantitative analysis (n = 3).

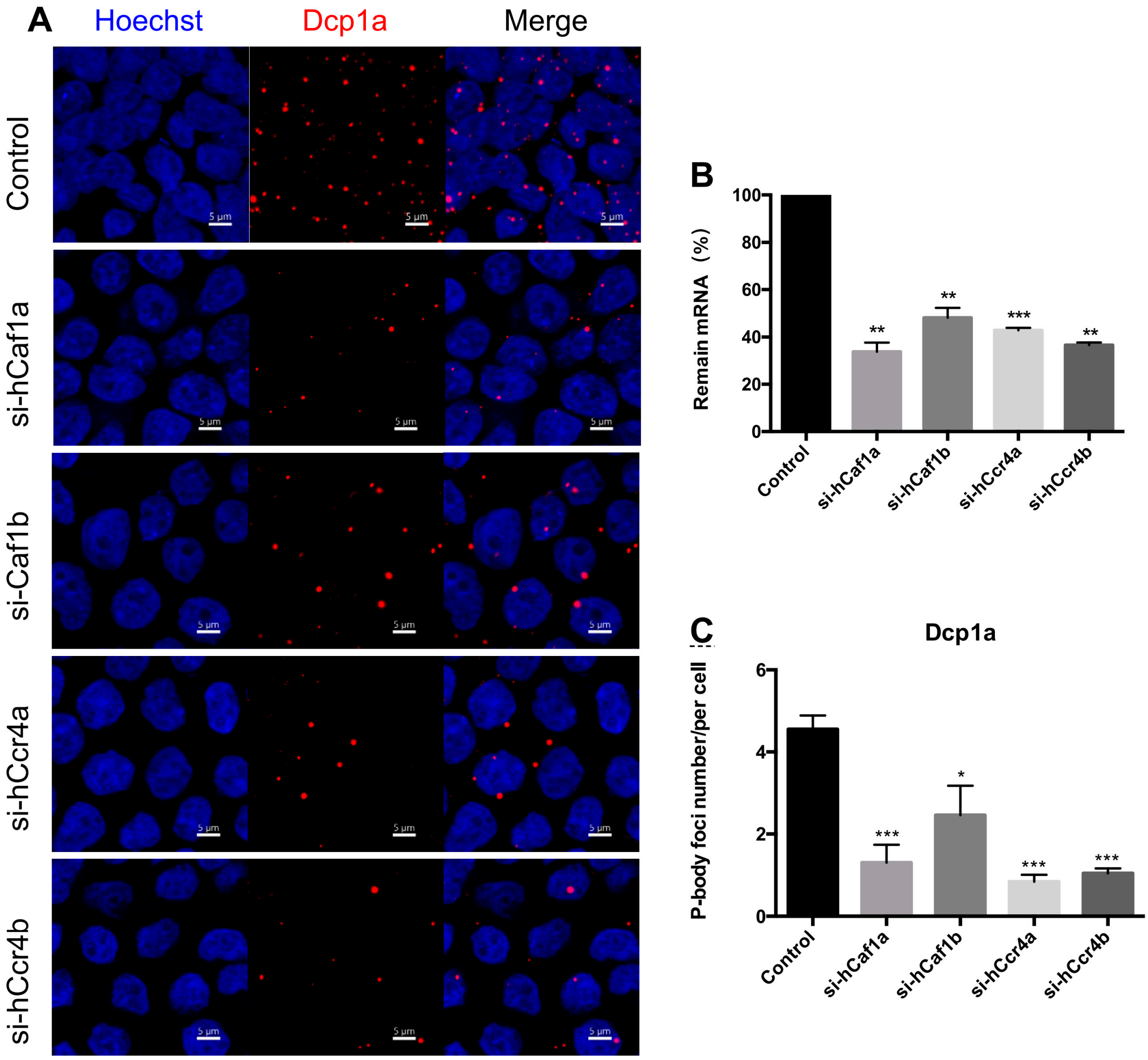

Figure S10. Effect of hCcr4a/b- or hCaf1a/b-knockdown on P-body formation in the HEK-293Tcells: (**A**) Representative confocal images of P-body formation visualized by two P-body marker protein Dcp1a; (**B**) Knockdown efficiency of the four genes evaluated by qPCR; (**C**) Quantitative analysis of the number of P-bodies per cell calculated from 10 random viewing fields for each repetition (n = 3).

A: Original western blot gels for Figure S3

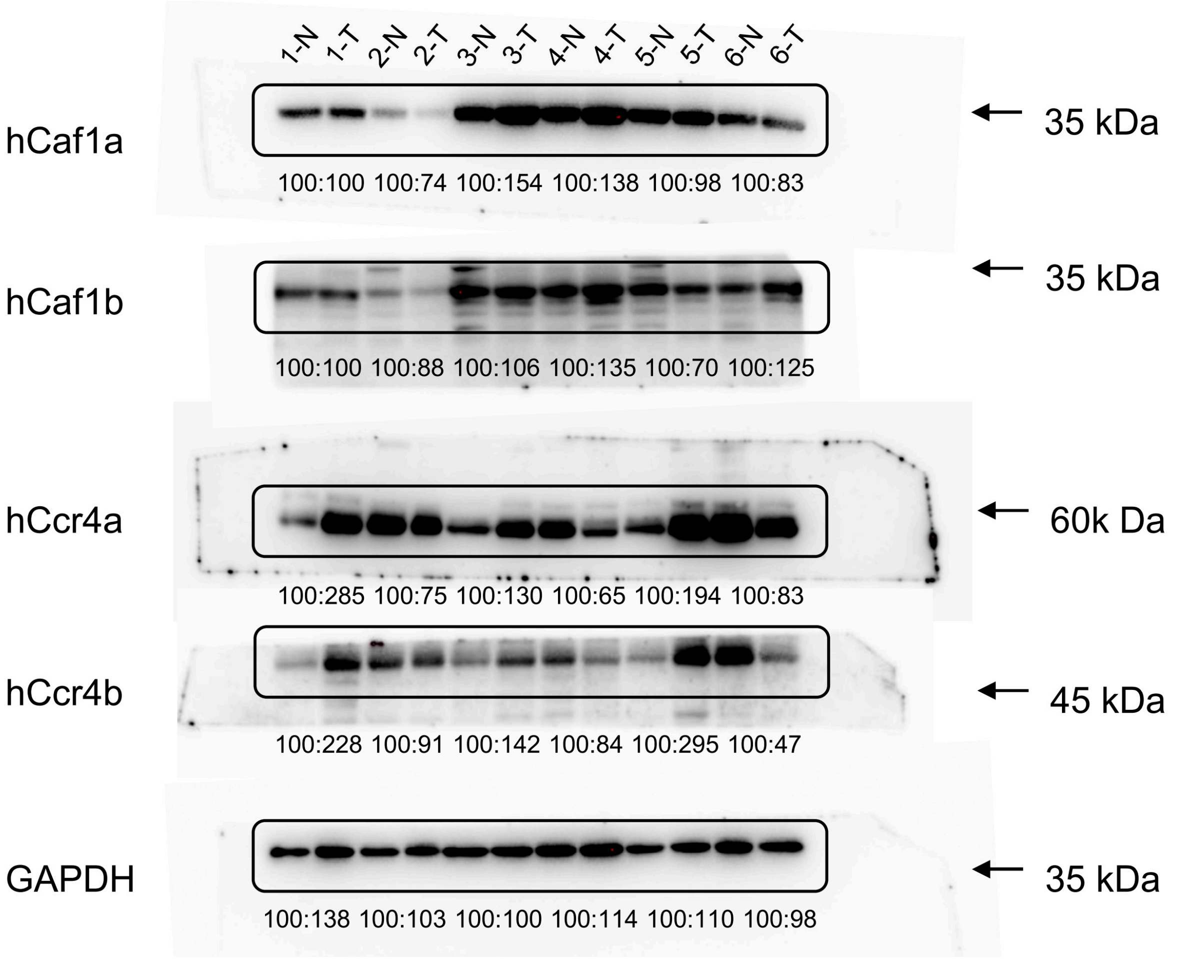

B: Original western blot gels for Figure S5

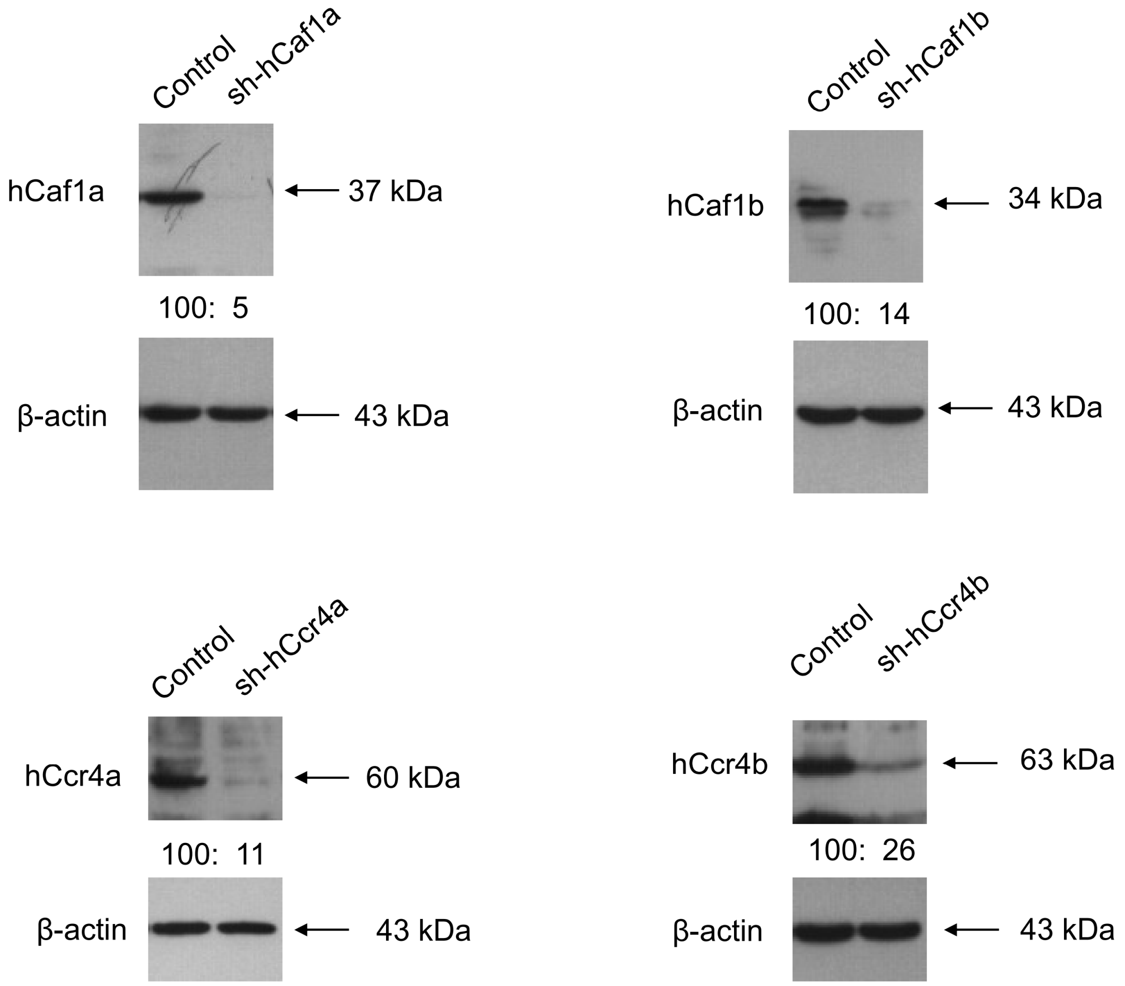

C: Original western blot gels for Figure S7

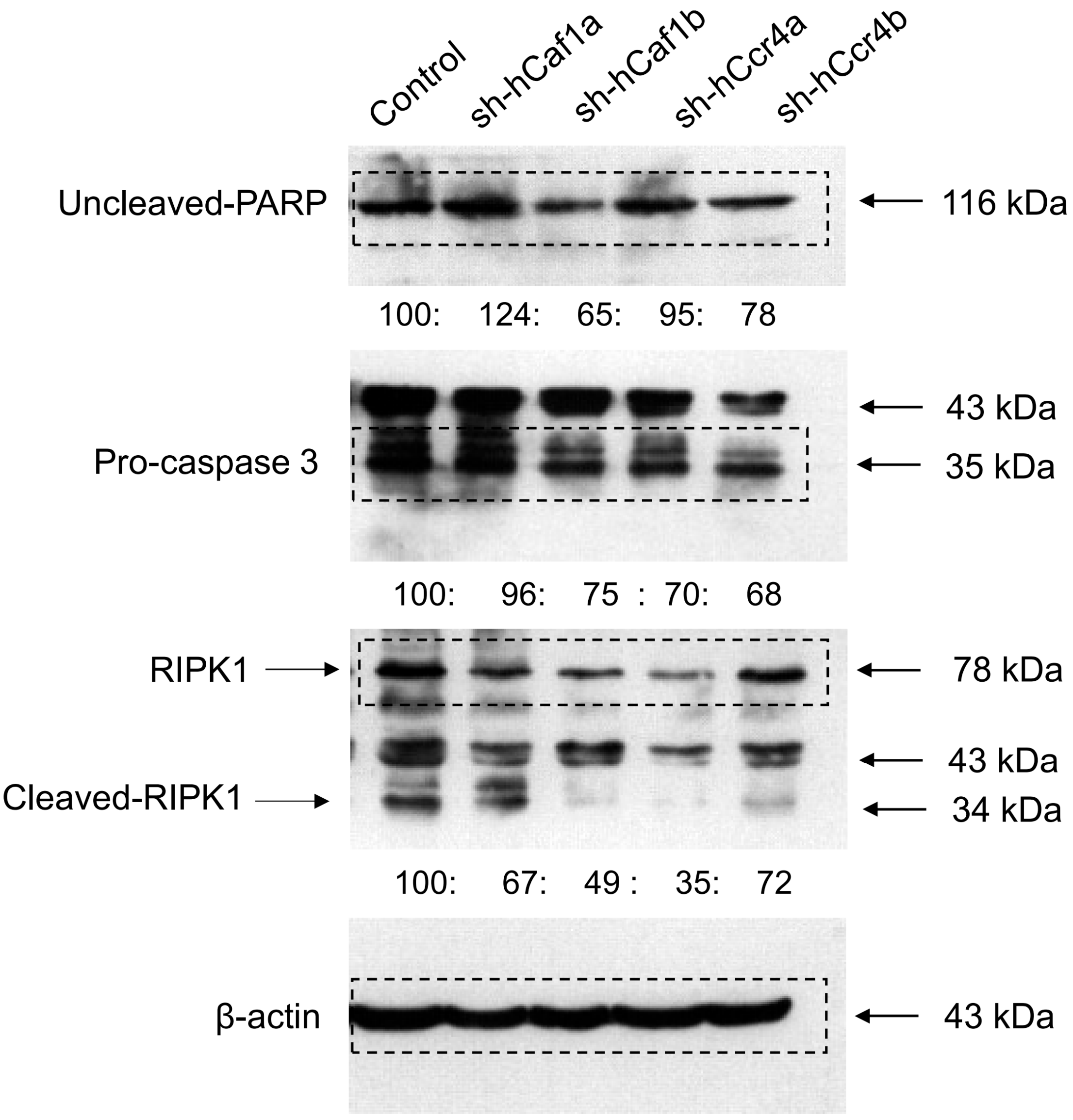

D: Original western blot gels for Figure S8

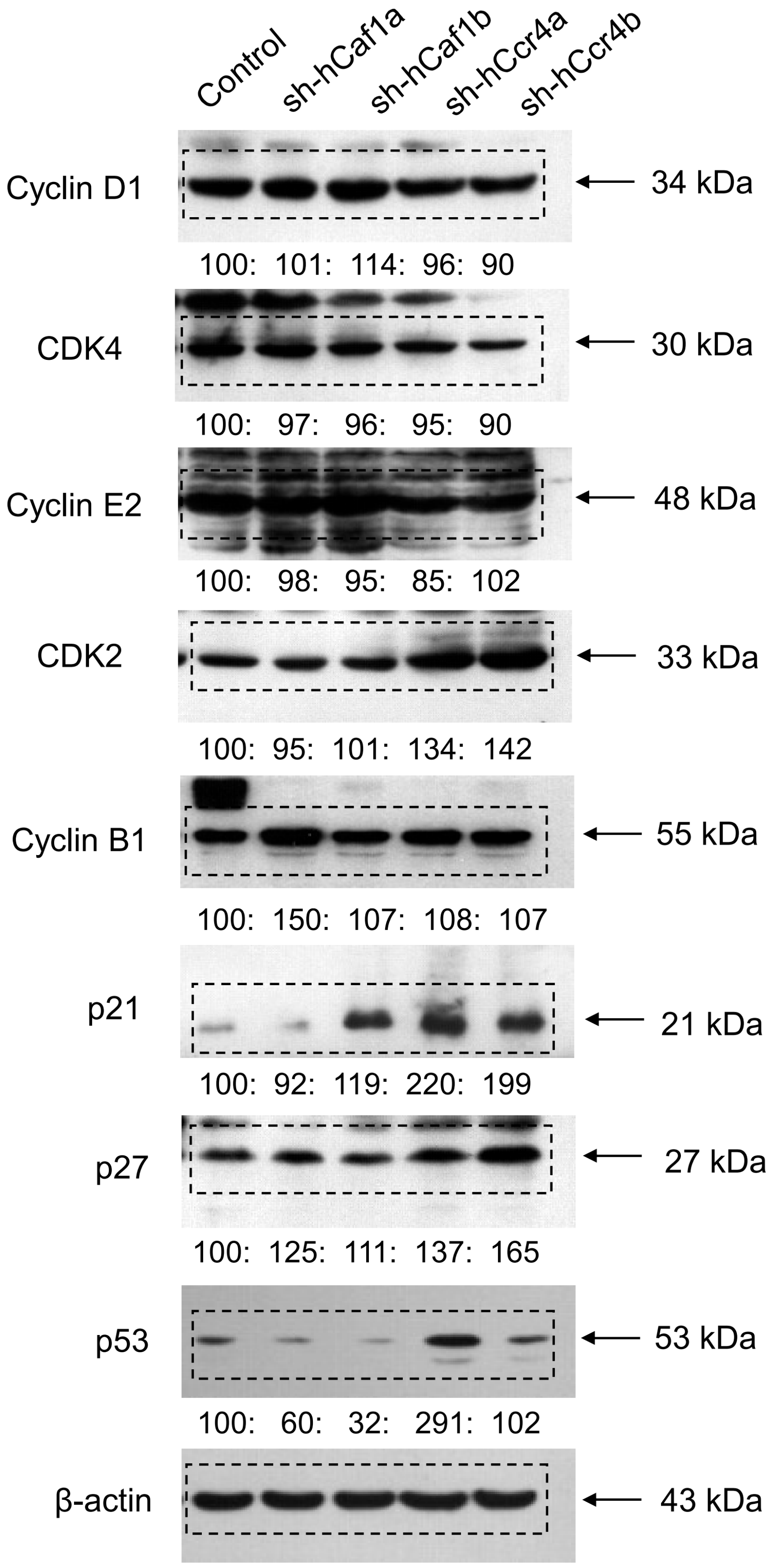

E: Original western blot gels for Figure S9

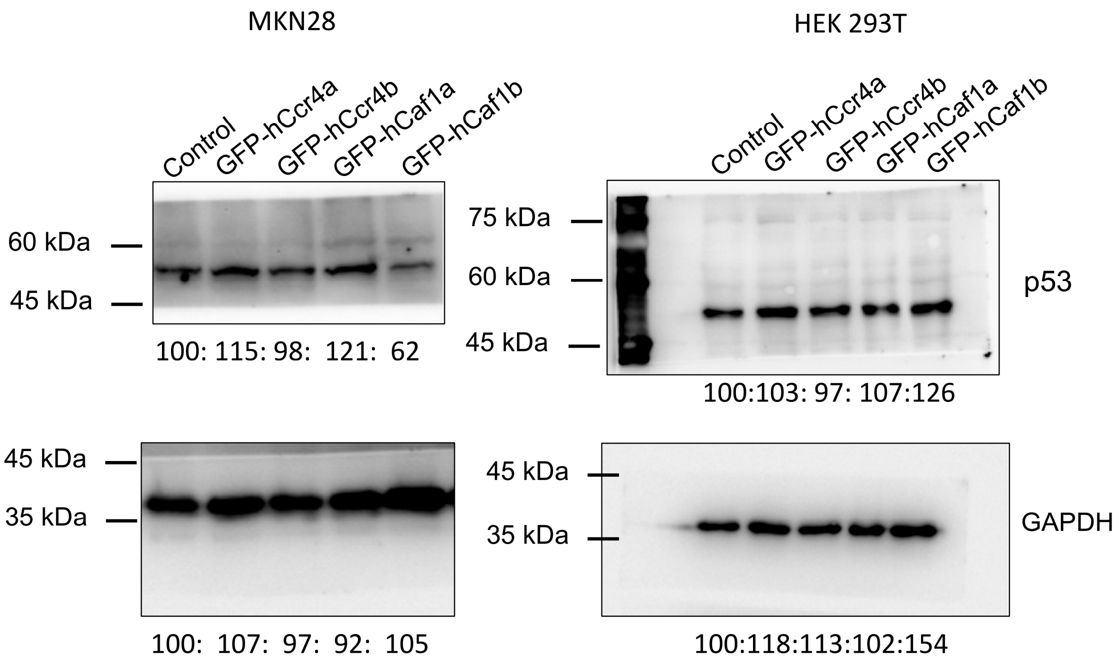

Figure S11. Original western blot gels.

Table S1. The shRNA sequences targeting hCaf1a, hCaf1b, hCcr4a and hCcr4b to generate stable knocking down cell lines.

shRNA	Sense (5′-3′)	Antisense (5′-3′)
sh-hCaf1a	GATCCGTGTAATGTAGACTTGTTAATT CAAGAGATTAACAAGTCTACATTACA CCTTTTTGGAAA	AGCTTTTCCAAAAAGGTGTAATGTA GACTTGTTAATCTCTTGAATTAACAAG TCTACATTACACG
sh-hCaf1b	GATCCGCTCAGTTACAGTTATATTCTC AAGAGAAATATAACTGTAACTGAGCA CTTTTTTGGAAA	AGCTTTTCCAAAAAAGTGCTCAGTTAC AGTTATATTTCTCTTGAGAATATAACT GTAACTGAGCG
sh-hCcr4a	GATCCGCCTGATGCCTTACACGAATTC AAGAGATTCGTGTAAGGCATCAGGCT TTTTTGGAAA	AGCTTTTCCAAAAAAGCCTGATGCCTT ACACGAATCTCTTGAATTCGTGTAAGG CATCAGGCG
sh-hCcr4b	GATCCGACCCAGAGTATTCTGATGTTC AAGAGACATCAGAATACTCTGGGTCT TTTTTGGAAA	AGCTTTTCCAAAAAAGACCCAGAGTA TTCTGATGTCTCTTGAACATCAGAATA CTCTGGGTCG
Negative control	GATCCACTACCGTTGTTATAGGTGTTC AAGAGACACCTATAACAACGGTAGTT TTTTGGA	

Table S2. The siRNA sequences targeting hCaf1a, hCaf1b, hCcr4a and hCcr4b.

siRNA	Sense (5′-3′)	Antisense (5′-3′)
si-hCaf1a	GCAAGACCCAUUGGAGAAUTT	AUUCUCCAAUGGGUCUUGCTT
si-hCaf1b	GGUGUUGUGGUGCGACCAATT	UUGGUCGCACCACAACACCTT
si-hCcr4a	CCGCAGAACUCGGAAACAUTT	AUGUUUCCGAGUUCUGCGGTT
si-hCcr4b	GGUGUUGUGGAAUACUUAATT	UUAAGUAUUCCACAACACCTT
Negtive Control	UUCUCCGAACGUGUCACGUTT	ACGUGACACGUUCGGAGAATT
